# Meal Frequency and Skipping Breakfast Are Associated with Chronic Kidney Disease

**DOI:** 10.3390/nu12020331

**Published:** 2020-01-27

**Authors:** Young Jin Kim, Jung Hwan Yoon, Hong Sang Choi, Chang Seong Kim, Eun Hui Bae, Seong Kwon Ma, Soo Wan Kim

**Affiliations:** Department of Internal Medicine, Chonnam National University Medical School, Gwangju 61469, Korea; vfrider@daum.net (Y.J.K.); sdewsss@naver.com (J.H.Y.); hongsang38@hanmail.net (H.S.C.); laminion@hanmail.net (C.S.K.); baedak@hanmail.net (E.H.B.)

**Keywords:** meal frequency, breakfast skipping, chronic kidney disease, Korea

## Abstract

Chronic underhydration and malnutrition can be associated with irreversible renal damage. This study investigated the association of meal frequency and breakfast skipping with chronic kidney disease (CKD) in South Korea. Participants (4370 participants from the Korean National Health and Nutrition Examination Survey VI 2013–2014) were divided into two groups based on meal frequency: ≥ 15 or < 15 meals/week. They were further divided into four groups based on the frequency of breakfast, lunch, and dinner consumed in the previous year. The data were analyzed with complex samples logistic regression. We found that 9.6% of the participants (*n* = 412) had CKD, which was associated with gender, body mass index, serum fasting glucose, daily calorie intake, hypertension, diabetes, and cerebrovascular accident. Participants consuming <15 meals/week had a higher risk of CKD than those who consumed ≥15 meals/week (adjusted odds ratio [OR] 1.531, 95% confidence interval [CI] 1.209–1.938). Participants who rarely had breakfast showed a higher risk of CKD than those who had breakfast 5–7 times/week (adjusted OR 1.572, 95% CI 1.108–2.231). Our findings suggest that <15 meals/week or skipping breakfast is associated with a higher risk of CKD in the general South Korean population, especially for men or persons aged 42–64 years.

## 1. Introduction

Chronic kidney disease (CKD) is defined as structural or functional abnormalities of the kidney that affect health for more than 3 months [1]. CKD is associated with cardiovascular disease, anemia, and metabolic bone disease, as well as adverse outcomes such as medication toxicity, infection, and even death [1,2,3]. It occurs in 11% of the population worldwide [2,3,4,5]. The prevalence of CKD, specifically, in South Korea is reported to be 7.9%–13.7%, and generally accepted to be 10.5% [6,7,8,9]. The reported increase in the incidence of CKD and end-stage kidney disease (ESKD) has greatly increased the associated socioeconomic costs for individuals and society [3,10,11]. CKD is associated with medication usage, chronic diseases, such as hypertension and diabetes, infectious diseases, race, socioeconomic status, and environmental factors [2,3].

Previous studies have suggested a positive association between increased hydration and preservation of kidney function. In addition, the limited evidence known about irreversible kidney damage from chronic underhydration and malnutrition also suggest a possible relationship between dietary patterns and CKD [12,13]. Studies have shown that low meal frequency and skipping breakfast are associated with an increased risk of coronary heart disease (CHD), metabolic syndrome, and diabetes [14,15,16,17,18,19,20,21,22,23,24,25,26]. A few previous reports have suggested that individuals with or without CKD should consume a specific diet according to the individual’s kidney health; nevertheless, the influence of dietary patterns on CKD has rarely been reported [27,28,29,30,31,32]. Chronic diseases such as hypertension and diabetes are associated with CKD, and if dietary patterns are associated with such chronic diseases, we can suspect that dietary patterns may be linked with CKD [2,3,14,15,16,17,18,19,20,21,22,23,24,25,26]. A few previous studies on the association between dietary patterns and CKD showed several limitations in the information of the frequency of meal or breakfast, the amount of calorie intake, the definition of dietary pattern, and the selection of study population [33,34,35,36,37]. Therefore, the aim of this study was to measure the association of meal frequency and breakfast skipping with the prevalence of CKD in the general Korean population.

## 2. Methods

### 2.1. Study Population

**Figure 1:**
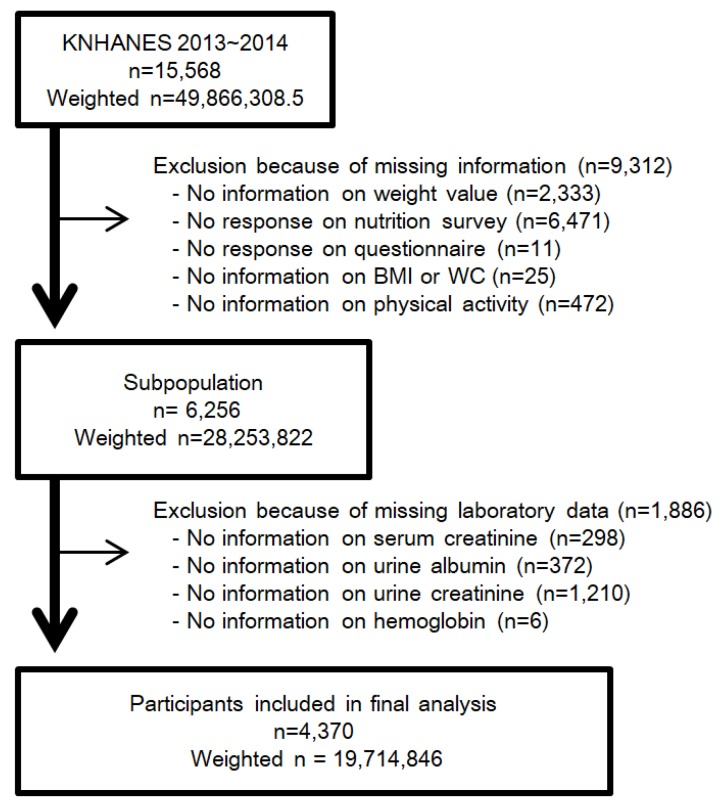
Flow diagram for determining study population with the exclusion and inclusion criteria. Abbreviation: KNHANES, Korean National Health and Nutrition Experimental Survey; *n*, unweighted number of participants; weighted *n*, weighted number of participants using complex samples design; BMI, body mass index; WC, waist circumference.

The Korean National Health and Nutrition Experimental Survey (KNHANES) is a nationwide, population-based, cross-sectional study of the health and nutritional status of South Korean residents. The Korea Centers for Disease Control and Prevention (KCDC) conducts the survey to gather sociodemographic information, anthropometric measurements, laboratory data, a health questionnaire, and nutrition information. The KNHANES data are publicly available [38]. Written informed consent was obtained from each participant in KNHANES at the time of enrollment. We performed this study in accordance with the Declaration of Helsinki. This study was approved by the IRB of Chonnam National University Hospital (IRB No. CNUH-EXP-2019-157). The target population of KNHANES was drawn using a multi-stage, stratified, and clustered probability sampling design. In each year, 192 primary sampling units (PSUs) were selected from about 200,000 geographically defined PSUs nationwide. A PSU includes about 60 households, and 20 households were systematically sampled from each PSU. In the sampled households, participants aged one year or above were selected. All the statistics of KNHANES were calculated using sample weights allocated to sample participants. The weights were set for sample participants to represent the whole Korean population. The weights based on the inverse of selection probabilities and inverse of response rates were modified by post-stratification with sex and age [39]. We retrospectively analyzed data of 15,568 participants from the KNHANES 2013–2014. After weighting by complex samples design, 15,568 participants representing 49,866,308.5 South Koreans were considered for the study [9,39,40,41]. According to Statistics Korea, the population of South Korea was 48.580 million in 2010, and 51.069 million in 2015 [42]. Participants aged below 19 years or older than 64 years were excluded, because the nutrition survey was implemented for participants aged between 19 and 64 years old. We excluded participants who did not answer the entire questionnaire or could not undergo the laboratory tests. A total of 4370 individuals representing 19,714,846 persons were included (Figure 1). This represents 39.5% of the total estimated Korean population, and 58.3% of the estimated population aged between 19 and 64.

### 2.2. Breakfast, Lunch, and Dinner Frequency and Meal Frequency

The frequency of breakfast was defined by the answer to the nutrition survey question, “How many breakfasts have you had a week in the last year?” The answers were: (1) 5–7 times/week; (2) 3–4 times/week; (3) 1–2 times/week; and (4) seldom (nearly 0 time/week). The participants were categorized into four groups according to their answer. In the same manner, the frequencies of lunch and dinner were defined by the question, "How many lunches (dinners) have you had a week in last the year?" The participants were categorized into four groups according to their answers for lunch and dinner, respectively [40,41,43]. Meal frequency was defined as the sum of the frequencies of breakfast, lunch, and dinner. Participants were divided into two groups according to ≥15 meals or <15 meals per week. 

### 2.3. Definition of CKD and Other Covariates

We defined CKD as an estimated glomerular filtration rate (eGFR) < 60 mL/min/1.73 m^2^, or a random urine albumin-creatinine ratio (ACR) ≥ 30 mg/g Cr [1]. eGFR was calculated using the Chronic Kidney Disease-Epidemiology Collaboration (CKD-EPI) equation [44]. The health interview and examinations were performed by well-trained nurses and the survey team in a health examination center built-in mobile vehicle. Blood pressure (BP) was measured three times after at least five minutes of rest in a stable sitting position according to the standardized protocol of the American Heart Association (AHA). The mean value of the second and third measurement was recorded as the final BP.

A questionnaire was employed to obtain sociodemographic information including age, gender, annual family income, education level, employment status, marital status, insurance, smoking, alcohol consumption, quality of life (EuroQol-5D), and physical activity [45]. Physical activity was defined as metabolic equivalent task (MET), calculated by intensity and time of activity at work or in leisure for the past seven days assessed using the International Physical Activity Questionnaire (IPAQ) [46]. Participants were grouped into three categories by MET scores (MET-minutes/week 0–599, 600–2999, and ≥3000).

Hypertension was defined as systolic BP ≥ 140 mmHg, diastolic BP ≥ 90 mmHg, usage of anti-hypertensive agents, or diagnosis by a physician. Diabetes mellitus was defined as fasting glucose level ≥ 126 mg/dL, glycated hemoglobin (HbA1c) ≥ 6.5%, usage of anti-diabetes medications, or diagnosis by a physician. Dyslipidemia was defined as total cholesterol ≥ 240 mg/dL, low-density lipoprotein (LDL) ≥ 160 mg/dL, high-density lipoprotein (HDL) < 40 mg/dL, triglyceride ≥ 200 mg/dL, usage of lipid lowering agent, or diagnosis by a physician. Medical history of coronary artery disease (CAD) and cerebrovascular accident (CVA) were defined according to a physician’s diagnosis. 

In accordance with the guideline from the nutritional survey in KNHANES VI, a food intake frequency table was used to examine the amount and frequency of food intake for 113 food items over the past year [47]. The methods and equipment to measure anthropometric and laboratory data are detailed in Supplementary Text S1.

### 2.4. Statistical Methods

We employed the complex samples analysis module in SPSS (version 23.0, SPSS Inc. Chicago, Il, USA). The sociodemographic and clinical variables were comparatively analyzed using the complex samples general linear model for continuous variables and the complex samples chi-square test for categorical variables. Unweighted number of samples and weighted mean value with 95% confidence interval (CI) were expressed for continuous variables. Unweighted number of samples and weighted percentage with 95% CI were represented for categorical variables. For each variable, complex sample univariate logistic regression was performed, and the proper model was set by selecting statistically significant and clinically important variables. To determine the associations of CKD with meal frequency and frequency of breakfast/lunch/dinner, complex samples multivariate logistic regression was performed for risk factors including gender, age, body mass index (BMI), hypertension, diabetes, history of CAD, CVA and smoking, hemoglobin, fasting glucose, HDL, LDL, and self-reported daily calorie intake. The result was expressed as odds ratio (OR) with 95% CI.

## 3. Results

### 3.1. Baseline Characteristics

**Figure 2:**
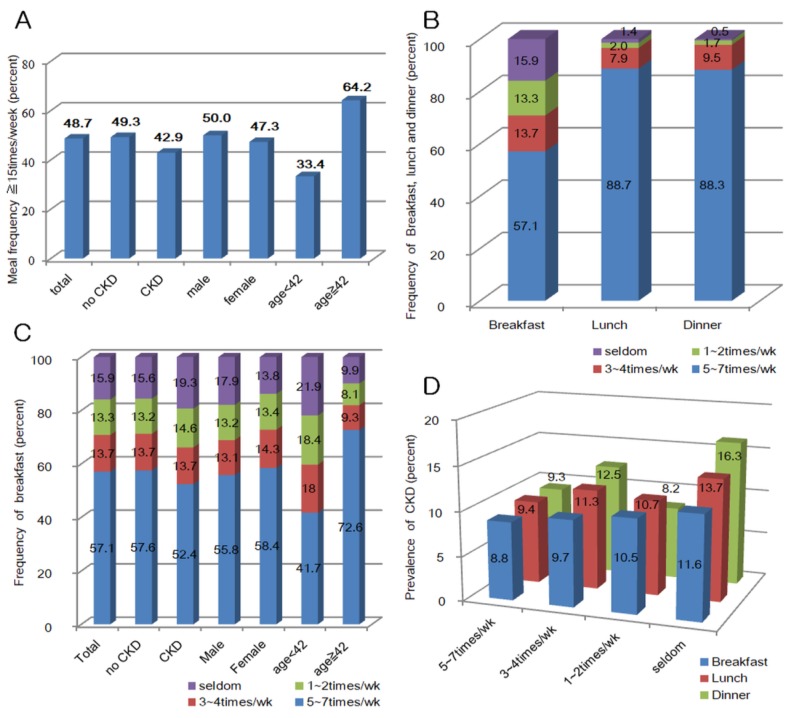
(**A**) Meal frequency at least ≥ 15 times/week; (**B**) frequency of breakfast, lunch and dinner; (**C**) frequency of breakfast by chronic kidney disease (CKD), gender, and age (years); and (**D**) prevalence of CKD by the frequency of breakfast, lunch, and dinner.

The prevalence of CKD was 9.6%, 9.4% of the population categorized as having CKD with albuminuria and 0.8% diagnosed by a decrement in eGFR. The prevalence of CKD was higher in males and in participants above the median age of 42 years (Supplementary Figure S1). In the previous one year, 48.7% of the participants reported a meal frequency of ≥15 times/week. The meal frequency of ≥15 per week was common among males, participants without CKD, or those above the median age (42 years). The rate of breakfast skipping was high: 57.1% participants ate breakfast 5–7 per week and 15.9% rarely had breakfast. By contrast, the frequency of skipping lunch or dinner was low: 88.7% and 88.3% of people consumed 5–7 lunches and dinners per week, respectively (Supplementary Figure S2). Age, gender, and CKD were also associated with the frequency of breakfast, lunch, and dinner. The prevalence of CKD increased as the frequency of breakfast, lunch, and dinner decreased (Figure 2).

The baseline characteristics of our study population are detailed in Supplementary Tables S1–S4. The baseline characteristics of participants who had greater than or fewer than 15 meals per week are summarized in Table 1. Participants who consumed < 15 meals/week were younger, more educated, smokers, married, had lower annual family income, and had fewer underlying chronic diseases, such as hypertension or diabetes, but showed a higher prevalence of CKD.

### 3.2. Association of CKD with Frequency of Meal

Participants with meal frequency < 15 times/week showed a higher adjusted OR (1.531, 95% CI 1.209–1.938) than individuals who consumed ≥ 15 meals per week (Table 2). Male gender, BMI lower than 18.5 kg/m^2^, hypertension, diabetes, previous history of CVA, high fasting glucose, and low daily calorie intake were risk factors for CKD. Persons who seldom had breakfast were at a higher risk for CKD (OR 1.572, 95% CI 1.108–2.231) than in the reference group who had breakfast 5–7 times/week (Table 3). The other two groups, who had breakfast 3–4 times per week or 1–2 times a week, showed increased ORs; however, the increase was not statistically significant. There was no significant association with the frequency of lunch or dinner and CKD (Supplementary Tables S5 and S6).

### 3.3. Association of CKD with Frequency of Meal Stratified by Gender and Age

**Figure 3:**
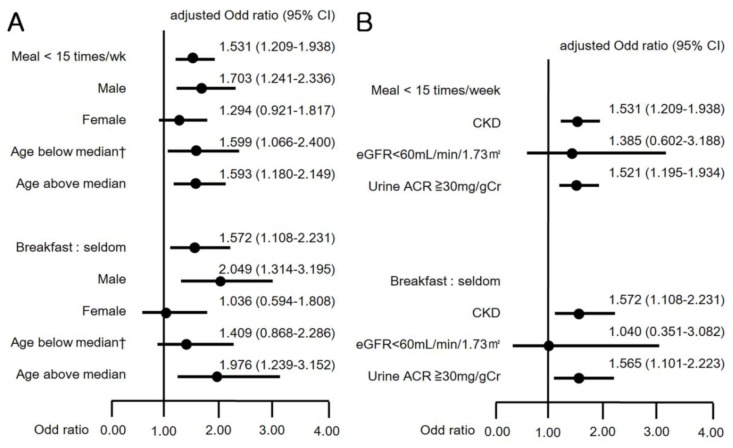
(**A**) Odds ratios (95% CI) for CKD by subgroup analysis with gender and age; and (**B**) Odds ratios (95% CI) for CKD by subgroup analysis with eGFR and urine albumin-creatinine ratio (ACR). † Old cerebrovascular accident not included in the analysis.

The association between CKD and dietary patterns was inconsistent after stratification by gender and median age of 42 years (Figure 3A). CKD was associated with meal frequency in men (OR 1.703, 95% CI 1.241–2.336), in those below the median age of 42 years (OR 1.599, 95% CI 1.066–2.400), and in those aged above the median age (OR 1.593, 95% CI 1.180–2.149), but not in women (OR 1.294, 95% CI 0.921–1.817). In the subgroup analysis for breakfast frequency, CKD was associated with breakfast skipping in men (OR 2.049, 95% CI 1.314–3.195) and in persons aged above the median age (OR 1.976, 95% CI 1.239–3.152) but not in women (OR 1.036, 95% CI 0.594–1.808), or in participants aged below the median age (OR 1.409, 95% CI 0.868–2.286). By contrast, the subgroup analysis considering the daily calorie intake showed that women had an association of low daily calorie intake with the prevalence of CKD, but men did not (Table 4).

### 3.4. Association of CKD with Frequency of Meal Stratified by eGFR and Urine ACR

We categorized separately the definition of CKD into a decrease in eGFR < 60 mL/min/1.73 m^2^ and an increment in urine ACR ≥ 30 mg/gCr. Then, we analyzed the variation in CKD prevalence with respect to meal frequency and breakfast skipping (Figure 3B). Fewer than 15 meals/week and skipping breakfast were significantly associated with the increment of urine ACR (OR 1.521, 95% CI 1.195–1.934; OR 1.565, 95% CI 1.101–2.223), but were not associated with the decrease in eGFR (OR 1.385, 95% CI 0.602–3.188; OR 1.040, 95% CI 0.351–3.082).

## 4. Discussion

To prevent CKD and its progression, previous studies have suggested healthy nutritional approaches, such as the DASH (Dietary Approaches to Stop Hypertension), the Mediterranean, or vegetarian diet [27,28,29,30,31,32]. Nevertheless, research on the association among meal frequency, breakfast skipping, and CKD prevalence is scarce. Two Japanese studies have shown that unhealthy lifestyle behaviors (late-night dinners and bedtime snacking) were related to CKD in middle-aged men; however, breakfast skipping was not. In these studies, breakfast skipping was defined as <4 breakfast per week, and the frequency of breakfast was not classified in more detail. In addition, there was no information on calorie intake. In one publication, the study was conducted only in men and the age of participants was 50.9 [8.0] years (mean (standard deviation)), which was relatively high with a narrow range. In another study, snacks after supper and skipping breakfast were combined as a less healthy eating pattern in the analysis [33,34]. Several other studies considered Ramadan fasting, in which Muslims do not consume food between sunrise and sunset during the ninth month of the Muslim calendar. During this month, they consume meals freely only after sunset. Previous studies on Ramadan fasting showed no significant deterioration of kidney function, but we could not identify the frequency of meal and the amount of calorie intake in these studies [35,36,37]. The AHA suggests that daily breakfast consumption can decrease the risk of harmful effects from glucose and insulin metabolism [14]. Alternate-day or intermittent fasting may be effective for weight loss, controlling dyslipidemia, and hypertension; nevertheless, their long-term effects are unclear [23,25,26]. Changing the meal frequency was not effective for weight loss or improving the risk for CHD without calorie restriction [14]. Many other studies have suggested the association of meal frequency and breakfast skipping with the risk of CHD, diabetes, and metabolic syndrome [15,16,17,18,19,20,21,22]. 

Here, we studied the effect of low weekly meal frequency and breakfast skipping over a one-year period to investigate the prevalence of CKD. Our results suggest a higher prevalence of CKD in participants who consumed < 15 meals or rarely had breakfast during a week. The major risk factors were male gender, low BMI, hypertension, diabetes, CVA, high fasting glucose, and low daily calorie intake. It is well documented that hypertension, diabetes, and metabolic syndrome are associated with CKD [48,49,50]. BMI higher than 25.0 kg/m^2^ or lower than 18.5 kg/m^2^ was statistically significant in a univariate analysis; however, after adjustment for hypertension, the significance of BMI higher than 25.0 kg/m^2^ disappeared. This finding was thought to be caused by high correlation between high BMI and hypertension. A BMI < 18.5 kg/m^2^ remained significant [51,52,53]. Physical activity did not showed an association with the prevalence of CKD. In our study, the results revealed little significance for CHD, probably because the prevalence of CHD was very low in the younger enrolled population. The association between breakfast skipping and CKD, and between <15 meals/week and CKD were quite similar. This finding is attributed to the 85.3% concordance between the groups who had 5–7 breakfasts/week and who had ≥15 meals/week. However, for lunch and dinner, the concordances were only 54.9% and 55.2%, respectively (Supplementary Figure S3). 

We found a few differences in the subgroup analyses by gender, age, and the categories of CKD by eGFR or albuminuria. Men showed a higher risk of CKD with low meal frequency; however, women did not. Men or persons aged above 42 years had higher risks of CKD due to breakfast skipping; however, women or persons below 42 years did not. Low meal frequency or breakfast skipping affected the prevalence of CKD as an increase in albuminuria, but not as a decrease in eGFR. 

In a predictive model study of survival rates during famines undertaken in various countries in the 19th and 20th centuries, women showed survival advantages ranging from 5% to 210%, although in some famines, no female advantage was identified [54]. Gender-specific differences in type, distribution, and function of adipocytes, secretion of adipokine, activation of the sympathetic nervous system in metabolic disease, and adipose tissue biology have been suggested recently [55,56]. In some murine models, female rats had lower energy consumption than males during calorie restriction, with gender-specific deactivation of brown adipose tissue [57]. A series of previous reports presented associations between cardiometabolic risk factors and eating frequency; showing significant results in men but not in women [15,20,21,22,24,33]. The subgroup analysis with daily calorie intake revealed that skipping breakfast and low meal frequency was associated with CKD in men; however, daily calorie intake was not. Women showed the opposite result. This contrast suggests a gender-specific difference in the metabolic response for dietary patterns and the level of daily energy consumption. 

Previous studies considered the association of breakfast skipping and meal frequency with CHD risk, diabetes, and metabolic syndrome, including longitudinal studies conducted for varying durations of two weeks to 25 years, either retrospective or prospective. We believe that skipping breakfast and low meal frequency for several years can manifest as CHD, diabetes, or metabolic syndrome [14,15,16,17,18,19,20,21,22,23,24,25,26]. CKD induced by these factors would take a considerable amount of time to develop. The present study had a cross-sectional design based on the dietary pattern of participants in the previous year preceding the survey. It was not a long-term follow-up study. Therefore, the association of CKD with breakfast skipping or low meal frequency was statistically significant in higher age groups that may have retained the dietary pattern for a longer time. It was not significant in the lower age group, which we believe is because this age group has maintained this dietary pattern for a relatively shorter time. In this context, we believe that CKD manifesting as a decline in eGFR did not show a significant association with dietary pattern, but rather as an increase of albuminuria, the step preceding decreases in eGFR. We hypothesize that, in middle-aged adults, low meal frequency or breakfast skipping may increase the risk of CHD, including hypertension, diabetes, and metabolic syndrome, thereby increasing the risk of CKD. Apart from this, the evidence is scarce and we cannot preclude another hypothesis that repeated short-term fasting and dehydration for long periods may increase the risk of CKD. Previous studies have provided the evidence for benefits of energy restriction for prevention of chronic disease and better clinical outcome. Wang et al. suggested a positive relationship between energy restriction and renal protection. However, most of the studies had been carried out on the murine models, and Wang pointed out the difficulties in performing clinical trials in humans [58]. In the studies, energy intake had been restricted, but water intake had not been limited. In our study, calorie intake was evaluated as the amount of food intake over the past year through self-reporting, and water intake could not be measured separately [10,11,12]. We believed that calorie intake should be accepted as the overall oral intake. Participants with low calorie intake might be with less overall oral intake and relatively less water intake. Clark et al. suggested that increasing water intake appeared to have some benefits on preserving renal function, by reducing vasopressin secretion [46]. Though there is very limited published evidence, Feehally and colleagues mentioned that chronic underhydration and malnutrition might cause irreversible renal damage by electrolyte imbalance, impaired acid-base balance, chronic rhabdomyolysis, Mesoamerican nephropathy, urinary tract stone, and infection [45,59,60].

Our study has several limitations. It is a cross-sectional study that cannot establish causal relationships between CKD and dietary patterns. Apart from the anthropometric and laboratory data, those of dietary patterns, nutrition survey, and medical and sociodemographic information were self-reported. Blood and urine were sampled only once, and the KNHANES questionnaire does not record information about medications. Consumption of snacks or consuming ≥4 meals per day was not checked.

Nevertheless, our study had some strengths. The KNHANES VI 2013–2014 provided a large, national-scale dataset, which was valuable for research. Its effectiveness is attributed to its representativeness for the general population rather than a specific patient group, and because it was conducted by a reliable national organization. Furthermore, the survey data contained a number of covariates in the questionnaire and laboratory data that could be processed to reduce potential confounders more effectively.

In conclusion, for Korean adults aged between 19 and 64, skipping breakfast or consuming fewer than 15 meals a week is significantly associated with the prevalence of CKD. The effect of dietary pattern shows differences in regard to gender and age groups. Evidence of the association between dietary patterns and kidney function is scarce; nevertheless, we gained small clues from this study. It is difficult to maintain diet-based interventions and observe the impact for long periods in a healthy population, therefore, we require further longitudinal prospective studies to confirm the effect of dietary patterns on CKD. Additional studies examining the effects of diet may accelerate the development of guidelines for recommended dietary patterns to prevent CKD and to promote kidney health awareness in the general population.

## Figures and Tables

**Table 1:** The concise baseline characteristics of population by meal frequency.

Variables	MF < 15		MF ≥ 15	
	*n* †	Mean or % (95% CI) ‡	*n* †	Mean or % (95% CI) ‡
Male sex (Yes or No)	822	51.1 (48.8–53.5)	982	53.9 (51.8–55.9)
Age (years)	2076	37.1 (36.5–37.8)	2294	45.6 (44.9–46.2)
Annual family income				
High	657	32.4% (29.4–35.6)	813	36.0 (32.8–39.4)
Medium high	661	31.5% (28.8–34.3)	710	31.9 (29.1–34.7)
Medium low	578	28.0% (25.4–30.8)	545	23.3 (20.8–26.1)
Low	175	7.8% (6.4–9.6)	221	8.5 (7.2–10.1)
Education				
More than college	907	44.8 (42.0–47.6)	830	38.9 (36.2–41.6)
High school	860	43.2 (40.7–45.9)	864	40.1 (37.7–42.6)
Middle school	153	6.4 (5.3–7.6)	268	10.7 (9.2–12.4)
Less than elementary school	154	5.5 (4.5–6.7)	330	10.3 (8.9–11.8)
Job status (Yes or No)	1358	67.9 (65.5–70.1)	1526	69.1 (66.8–71.3)
Marital status				
Married	1379	60.4 (57.4–63.3)	1887	78.0 (75.7–80.2)
Divorce or widowed	145	5.1 (4.2–6.3)	154	5.7 (4.7–6.9)
Not married	549	34.3 (31.4–37.4)	250	16.2 (14.2–18.4)
Smoking				
Never	1235	53.2 (50.7–55.7)	1489	58.7 (56.5–60.8)
Ex-smoker	288	15.3 (13.6–17.2)	428	20.7 (18.9–22.6)
Current	553	31.5 (29.1–34.0)	377	20.6 (18.4–23.0)
EuroQol-5D (*n*)	2076	0.966 (0.963–0.970)	2294	0.964 (0.959–0.968)
Systolic BP (mmHg)	2074	113.5 (112.7–114.2)	2294	115.8 (115.0–116.6)
Diastolic BP (mmHg)	2074	75.3 (74.8–75.9)	2294	76.2 (75.6–76.7)
Waist circumference (cm)	2076	79.9 (79.4–80.4)	2294	80.8 (80.3–81.3)
BMI (kg/㎡)	2076	23.7 (23.5–23.9)	2294	23.7 (23.6–23.9)
Physical activity (MET-minutes/week)	2076	1884 (1734–2031)	2294	1841 (1702–1981)
Serum creatinine (mg/dL)	2076	0.848 (0.835–0.862)	2294	0.850 (0.842–0.859)
CKD-EPI eGFR (mL/min/1.73 m^2^)	2076	102.8 (102.0–103.6)	2294	96.7 (95.9–97.5)
Random urine ACR (mg/gCr)	2076	29.7 (12.4–47.0)	2294	19.8 (12.8–26.7)
CKD (Yes or No)	206	10.7 (9.2–12.3)	206	8.5 (7.3–9.8)
Fasting glucose (mg/dL)	2076	95.2 (94.3–96.0)	2294	98.6 (97.6–99.6)
HbA1c (%)	2076	5.598 (5.568–5.629)	2294	5.784 (5.748–5.821)
HDL (mg/dL)	2076	51.7 (51.1–52.3)	2294	51.1 (50.5–51.6)
LDL (mg/dL)	2076	111.4 (109.8–113.1)	2294	112.5 (111.1–114.0)
Hemoglobin (g/dL)	2076	14.36 (14.29–14.43)	2294	14.36 (14.29–14.43)
Daily Calorie intake (kcal/day)	2076	2195 (2145–2244)	2294	2199 (2159–2238)
Hypertension (Yes or No)	323	14.4 (12.7–16.4)	582	23.8 (21.8–25.9)
Diabetes (Yes or No)	123	5.1 (4.2–6.1)	280	11.0 (9.6–12.5)
Old Coronary arterial disease (Yes or No)	16	0.6 (0.3–1.0)	34	1.1 (0.8–1.6)
Old cerebrovascular accident (Yes or No)	15	0.7 (0.4–1.3)	38	1.3 (0.8–2.0)
Dyslipidemia, (Yes or No)	685	33.2 (31.0–35.5)	880	37.4 (35.1–39.7)

Abbreviation: CI, confidence interval; BP, blood pressure; BMI, body mass index; MET, metabolic equivalent task; CKD-EPI, Chronic Kidney Disease-Epidemiology Collaboration; eGFR, estimated glomerular filtration rate; ACR, albumin creatinine ratio; CKD, chronic kidney disease; HbA1c, glycated hemoglobin; HDL, high-density lipoprotein; LDL, low-density lipoprotein. MF < 15: meal frequency < 15 times/week (*n* = 2076, weighted *n* = 10,113,047.5). MF ≥ 15: meal frequency ≥ 15 times/week (*n* = 2294, weighted *n* = 9601,798.5). †: unweighted *n*. ‡: weighted mean value or percentage with 95% confidence interval by complex samples analysis.

**Table 2:** The complex samples multivariate logistic regression for CKD by meal frequency.

Variables	Unadjusted OR (95% CI)	Model 1 (95% CI)	Model 2 (95% CI)	Model 3 (95% CI)
Meal frequency, <15/week (Ref.: ≥15 times/week)	1.294 (1.038–1.612)	1.531 (1.228–1.909)	1.634 (1.297–2.060)	1.531 (1.209–1.938)
Male gender (Yes or No)		1.418 (1.128–1.783)	1.370 (0.942–1.992)	1.877 (1.229–2.866)
Age (per year)		1.019 (1.009–1.029)	1.002 (0.991–1.013)	0.994 (0.982–1.006)
BMI (Ref.: normal, 18.5–25.0 Kg/m^2^)				
Obese (≥25.0 kg/m^2^)			1.241 (0.956–1.611)	1.258 (0.966–1.636)
Underweight (<18.5 kg/m^2^)			1.934 (1.068–3.503)	2.048 (1.120–3.745)
Physical activity (Ref.: high, ≥3000)				
Moderate (600–2999)			1.250 (0.897–1.742)	1.234 (0.884–1.722)
Low (<600)			1.069 (0.764–1.497)	1.021 (0.726–1.435)
Hypertension (Yes or No)			1.996 (1.498–2.660)	2.048 (1.536–2.730)
Diabetes (Yes or No)			3.210 (2.314–4.451)	1.924 (1.226–3.020)
Old CAD (Yes or No)			1.400 (0.662–2.960)	1.664 (0.793–3.492)
Old CVA (Yes or No)			2.066 (1.128–3.783)	2.199 (1.204–4.015)
Smoking (Ref.: never)				
Ex-smoker			0.911 (0.607–1.370)	0.906 (0.603–1.361)
Current smoker			0.823 (0.547–1.240)	0.846 (0.558–1.282)
Hemoglobin (g/dL)				0.910 (0.810–1.023)
Fasting glucose (mg/dL)				1.011 (1.005–1.017)
HDL (mg/dL)				1.004 (0.993–1.015)
LDL (mg/dL)				1.003 (1.000–1.007)
Daily calorie intake (g/day, log transformed)				0.772 (0.618–0.965)

Abbreviation: OR, odd ratio; Ref., reference; CAD, coronary artery disease; CVA, cerebrovascular accident. Model 1: gender, age. Model 2: Model 1 + BMI, physical activity (MET), hypertension, diabetes, old CAD, old CVA, smoking. Model 3: Model 2 + hemoglobin, fasting glucose, HDL, LDL, daily calorie intake.

**Table 3:** The complex samples multivariate logistic regression for CKD by breakfast frequency.

Variables	Unadjusted OR (95% CI)	Model 1 (95% CI)	Model 2 (95% CI)	Model 3 (95% CI)
Breakfast (Ref.: 5~7 times/week)				
3~4 times/week	1.106 (0.797–1.535)	1.288 (0.928–1.788)	1.353 (0.964–1.900)	1.339 (0.951–1.885)
1~2 times/week	1.220 (0.876–1.699)	1.438 (1.020–2.026)	1.525 (1.067–2.178)	1.413 (0.979–2.038)
Seldom	1.360 (0.982–1.882)	1.562 (1.114–2.191)	1.695 (1.199–2.397)	1.572 (1.108–2.231)
Male gender (Yes or No)		1.379 (1.097–1.733)	1.324 (0.912–1.920)	1.837 (1.206–2.800)
Age (per year)		1.018 (1.008–1.029)	1.001 (0.989–1.013)	0.993 (0.981–1.006)
BMI (Ref.: normal, 18.5–25.0 kg/m^2^)				
Obese (≥25.0 kg/m^2^)			1.263 (0.974–1.637)	1.278 (0.984–1.660)
Underweight (<18.5 kg/m^2^)			1.899 (1.046–3.449)	2.020 (1.102–3.703)
Physical activity (Ref.: high, ≥3000)				
Moderate (600–2999)			1.242 (0.892–1.730)	1.225 (0.878–1.708)
Low (<600)			1.056 (0.756–1.474)	1.007 (0.718–1.413)
Hypertension (Yes or No)			1.994 (1.494–2.661)	2.047 (1.533–2.734)
Diabetes (Yes or No)			3.194 (2.306–4.423)	1.913 (1.221–2.997)
Old CAD (Yes or No)			1.371 (0.647–2.904)	1.635 (0.778–3.437)
Old CVA (Yes or No)			2.041 (1.112–3.745)	2.173 (1.190–3.969)
Smoking (Ref.: never)				
Ex-smoker			0.907 (0.605–1.362)	0.902 (0.601–1.355)
Current smoker			0.824 (0.548–1.239)	0.849 (0.561–1.285)
Hemoglobin (g/dL)				0.907 (0.807–1.019)
Fasting glucose (mg/dL)				1.011 (1.005–1.017)
HDL (mg/dL)				1.004 (0.993–1.015)
LDL (mg/dL)				1.003 (1.000–1.007)
Daily calorie intake (g/day, log transformed)				0.767 (0.613–0.959)

**Table 4:** Subgroup analysis using the complex samples multivariate logistic regression for CKD by meal frequency, breakfast frequency, and self-reported daily calorie intake.

Variables	Unadjusted OR (95% CI)	Model 3 (95% CI)	Daily Calorie Intake (log Transformed)		*p* for Interaction ‡
			Unadjusted OR (95% CI)	Model 3 (95% CI)	
Overall					
Meal < 15 times/week	1.294 (1.038–1.612)	1.531 (1.209–1.938)	0.810 (0.659–0.996)	0.772 (0.618–0.965)	0.539
Breakfast, seldom	1.360 (0.982–1.882)	1.572 (1.108–2.231)		0.767 (0.613–0.959)	0.502
Male gender					
Meal < 15 times/week	1.229 (0.909–1.661)	1.703 (1.241–2.336)	0.712 (0.496–1.023)	0.869 (0.596–1.265)	0.621
Breakfast, seldom	1.388 (0.932–2.066)	2.049 (1.314–3.195)		0.880 (0.600–1.291)	0.551
Female gender					
Meal < 15 times/week	1.424 (1.042–1.945)	1.294 (0.921–1.817)	0.657 (0.491–0.880)	0.673 (0.500–0.905)	0.430
Breakfast, seldom	1.221 (0.731–2.039)	1.036 (0.594–1.808)		0.663 (0.493–0.891)	0.246
Age, under 42 years					
Meal < 15 times/week	1.633 (1.099–2.424)	1.599 (1.066–2.400) †	0.770 (0.553–1.073)	0.728 (0.512–1.035) †	0.987
Breakfast, seldom	1.391 (0.862–2.245)	1.409 (0.868–2.286) †		0.718 (0.506–1.020) †	0.929
Age, above 42 years					
Meal < 15 times/week	1.333 (1.020–1.743)	1.593 (1.180–2.149)	0.914 (0.695–1.202)	0.830 (0.631–1.091)	0.177
Breakfast, seldom	1.776 (1.133–2.784)	1.976 (1.239–3.152)		0.823 (0.623–1.088)	0.842

† Old CVA is not included in model 3, because the prevalence of old CVA is small in population under 42 years of age (old CVA: *n* = 4, weighted *n* = 21,566.5; population under 42 years of age: *n* = 1763, weighted *n* = 9,384,170). ‡ *p* for interactions between the frequency of meal/breakfast and daily calorie intake.

## Supplementary Materials

The following are available online at https://www.mdpi.com/2072-6643/12/2/331/s1, Text S1. Details of methods and equipment used for measuring the anthropometric and laboratory data, Table S1. Baseline characteristics of the study population by meal frequency, Table S2. Baseline characteristics of the study population by breakfast frequency, Table S3. Baseline characteristics of the study population by lunch frequency, Table S4. Baseline characteristics of the study population by dinner frequency, Table S5. Complex samples multivariate logistic regression for analyzing the prevalence of chronic kidney disease (CKD) by lunch frequency, Table S6. Complex samples multivariate logistic regression for analyzing the prevalence of CKD by dinner frequency, Figure S1. (A) Prevalence of Chronic kidney disease (CKD) in the study population; (B) Prevalence of CKD by gender; and (C) Prevalence of CKD by the median age of 42 years. Figure S2. (A) Frequency of lunch intake by chronic kidney disease (CKD), gender, and age, (B) Frequency of dinner intake by CKD, gender, and age, Figure S3. Association of meal frequency with the frequency of breakfast, lunch, and dinner.
