# Supplementary material for: Meal Frequency and Skipping Breakfast Are Associated with Chronic Kidney Disease
Source: Nutrients. 2020 Jan 27;12(2):331. doi: 10.3390/nu12020331 (PMC7071178; doi:10.3390/nu12020331)
Supplement: Supplementary file 1 [file nutrients-12-00331-s001.pdf]

nutrients

Meal Frequency and Skipping Breakfast Are Associated with Chronic Kidney Disease

Young Jin Kim, Jung Hwan Yoon, Hong Sang Choi, Chang Seong Kim, Eun Hui Bae, Seong Kwon Ma *,† and Soo Wan Kim *,†

Department of Internal Medicine, Chonnam National University Medical School, Gwangju 61469, Korea; vfrider@daum.net (Y.J.K.); sdewsss@naver.com (J.H.Y.); hongsang38@hanmail.net (H.S.C.); laminion@hanmail.net (C.S.K.); baedak@hanmail.net (E.H.B.)

* Correspondence: drmsk@hanmail.net (M.S.K); skimw@chonnam.ac.kr (S.W.K); Tel.: +82-62-220-6579 (M.S.K), +82-62-220-6271 (S.W.K); Fax.: +82-62-225-8578 (M.S.K. & S.W.K)

† These authors have contributed equally to this manuscript as correspondence authors.

Supplementary Materials

Supplementary Text S1. Details of methods and equipment used for measuring the anthropometric and laboratory data.

Supplementary Table S1. Baseline characteristics of the study population by meal frequency.

Supplementary Table S2. Baseline characteristics of the study population by breakfast frequency.

Supplementary Table S3. Baseline characteristics of the study population by lunch frequency.

Supplementary Table S4. Baseline characteristics of the study population by dinner frequency.

Supplementary Table S5. Complex samples multivariate logistic regression for analyzing the prevalence of chronic kidney disease (CKD) by lunch frequency.

Supplementary Table S6. Complex samples multivariate logistic regression for analyzing the prevalence of CKD by dinner frequency.

Supplementary Figure S1. (**A**) Prevalence of Chronic kidney disease (CKD) in the study population; (**B**) Prevalence of CKD by gender; and (**C**) Prevalence of CKD by the median age of 42 years. Abbreviation: eGFR, estimated glomerular filtration rate; ACR, albumin-creatinine ratio.

Supplementary Figure S2. (**A**) Frequency of lunch intake by CKD, gender, and age, (**B**) Frequency of dinner intake by CKD, gender, and age.

Supplementary Figure S3. Association of meal frequency with the frequency of breakfast, lunch, and dinner.

Supplementary Text S1. Details of methods and equipment used for measuring the anthropometric and laboratory data.

Blood pressure was measured using a mercury sphygmomanometer (Baumanometer Wall Unit 33(0850), Baum, Copiague, NY, USA). Body weight, height, and waist circumference were measured using an electronic scale (GL-6000-20, G-tech International, Uijeongbu, Korea), a height measure unit (Seca 225, Seca, Hamburg, Germany), and a tape measure (Seca 200), respectively. Participants were wearing thin health examination gowns without shoes.

Blood was sampled after 8 hours of fasting. Random urine was sampled using 20–30 mL of the first mid-stream urine in the morning. Samples were centrifuged and refrigerated immediately and transported to the central laboratory center. Serum and random urine creatinine were measured using the Jaffe rate-blanked and compensated methods using a Hitachi Automatic Analyzer 7600-210 (Hitachi, Tokyo, Japan), based on isotope dilution mass spectrometry (IDMS). The random urine albumin was measured using a turbidimetric assay using Hitachi Automatic Analyzer 7600 (Hitachi, Tokyo, Japan). Total cholesterol and triglyceride were measured using an enzymatic method, and high-density lipoprotein (HDL) and low-density lipoprotein (LDL) was measured using the homogeneous enzymatic colorimetric method. Serum fasting glucose was measured using the hexokinase-UV method. Random urine sodium was measured using the ion-selective electrode (ISE) method. These measurements were performed using the Hitachi Automatic Analyzer 7600-210 (Hitachi). HbA1c was measured with high performance liquid chromatography using Tosoh G8 (Tosoh Bioscience, Tokyo, Japan). Hemoglobin was measured using the sodium lauryl sulfate hemoglobin detection method on the XE-2100D differential analyzer (Sysmex, Kobe, Japan). Vitamin D was measured by radioimmunoassay using a 1470 WIZARD Gamma counter (Perkin Elmer, Turku, Finland). Hepatitis B surface antigen was measured with electrochemiluminescence immunoassay (ECLIA) using the Cobas 8000 e602 (Roche Diagnostics, Mannheim, Germany). Hepatitis C virus RNA was measured by a real-time PCR using Cobas AmpliPreq/Cobas TaqMan system (Roche). Participants self-reported information on body weight change, efforts for weight control, and dietary therapy within the past year. Depression was defined as Patient Health Questionnaire-9 score (PHQ-9) ≥10 or diagnosis by a physician. Medical history of malignancy, arthritis, tuberculosis, chronic obstructive pulmonary disease, allergic disease, thyroid disease and liver disease were defined according to a physician's diagnosis.

84 **Supplementary Table S1.** Baseline characteristics of the study population by meal frequency.

Variables	Meal frequency (n=4,370, weighted n=19,714,846)			
	MF <15		MF ≥15	
	n†	Mean or % (95% CI) ‡	n†	Mean or % (95% CI)‡
Male sex (Yes or No)	822	51.1 (48.8-53.5)	982	53.9 (51.8-55.9)
Age (years)	2,076	37.1 (36.5-37.8)	2,294	45.6 (44.9-46.2)
Annual family income				
High	657	32.4% (29.4-35.6)	813	36.0 (32.8-39.4)
Medium high	661	31.5% (28.8-34.3)	710	31.9 (29.1-34.7)
Medium low	578	28.0% (25.4-30.8)	545	23.3 (20.8-26.1)
Low	175	7.8% (6.4-9.6)	221	8.5 (7.2-10.1)
Education				
More than college	907	44.8 (42.0-47.6)	830	38.9 (36.2-41.6)
High school	860	43.2 (40.7-45.9)	864	40.1 (37.7-42.6)
Middle school	153	6.4 (5.3-7.6)	268	10.7 (9.2-12.4)
Less than elementary school	154	5.5 (4.5-6.7)	330	10.3 (8.9-11.8)
Job status (Yes or No)	1,358	67.9 (65.5-70.1)	1,526	69.1 (66.8-71.3)
Marital status				
married	1,379	60.4 (57.4-63.3)	1,887	78.0 (75.7-80.2)
divorce or widowed	145	5.1 (4.2-6.3)	154	5.7 (4.7-6.9)
not married	549	34.3 (31.4-37.4)	250	16.2 (14.2-18.4)
Insurance				
NHIS	1,992	95.9 (94.4-97.0)	2,244	98.0 (97.0-98.6)
medical aid	66	3.0 (2.0-4.3)	38	1.5 (1.0-2.3)
Personal insurance (Yes or No)	1,797	86.1 (84.1-87.8)	1,982	86.8 (84.6-88.6)
Smoking				
never	1,235	53.2 (50.7-55.7)	1,489	58.7 (56.5-60.8)
exsmoker	288	15.3 (13.6-17.2)	428	20.7 (18.9-22.6)
current	553	31.5 (29.1-34.0)	377	20.6 (18.4-23.0)
Alcohol consumption, ever	1,950	94.5 (93.2-95.6)	2,067	92.5 (91.3-93.5)
Alcohol consumption in last 1 year	1,303	66.4 (64.0-68.6)	1,189	56.6 (54.2-59.0)
EuroQol-5D (n)	2,076	0.966 (0.963-0.970)	2,294	0.964 (0.959-0.968)

Variables	Meal frequency (n=4,370, weighted n=19,714,846)			
	MF <15		MF ≥15	
	n†	Mean or % (95% CI) ‡	n†	Mean or % (95% CI)‡
Systolic BP (mmHg)	2,074	113.5 (112.7-114.2)	2,294	115.8 (115.0-116.6)
Diastolic BP (mmHg)	2,074	75.3 (74.8-75.9)	2,294	76.2 (75.6-76.7)
Waist circumference (cm)	2,076	79.9 (79.4-80.4)	2,294	80.8 (80.3-81.3)
Abdominal obesity (Yes or No)	217	13.3 (11.7-15.2)	271	14.3 (12.6-16.2)
BMI (kg/㎡)	2,076	23.7 (23.5-23.9)	2,294	23.7 (23.6-23.9)
Obesity in BMI				
Normal to pre-obesity (18.5~25)	1,317	62.1 (59.6-64.4)	1,486	65.1 (62.8-67.3)
Normal (18.5~23)	863	40.4 (37.9-42.8)	910	39.5 (37.3-41.7)
Pre-obesity (23~25)	454	21.7 (19.7-23.8)	576	25.6 (23.7-27.6)
Obesity (≥25)	657	32.9 (30.7-35.3)	727	31.5 (29.3-33.7)
1st obesity (25~30)	553	27.5 (25.4-29.8)	644	27.5 (25.5-29.6)
2nd obesity (30~35)	92	4.8 (3.9-6.0)	76	3.8 (2.9-4.9)
3rd obesity (≥35)	12	0.5 (0.3-1.0)	7	0.2 (0.1-0.6)
Under weighted (<18.5)	102	5.0 (4.0-6.2)	81	3.4 (2.6-4.4)
Exercise (Yes or No)	925	47.4 (44.5-50.2)	1,077	49.8 (47.3-52.3)
More than 150 mins/week	678	35.3 (32.7-38.1)	795	37.0 (34.7-39.4)
Physical activity (MET-minutes/week)	2,076	1,884 (1,734-2,031)	2,294	1,841 (1,702-1,981)
High (≥3000)	352	18.9 (17.0-20.9)	393	18.4 (16.6-20.3)
Moderate (600-3000)	889	43.7 (41.2-46.2)	986	43.7 (41.4-46.0)
Low (<600)	835	37.5 (35.0-40.0)	915	38.0 (35.6-40.4)
Serum creatinine (mg/dL)	2,076	0.848 (0.835-0.862)	2,294	0.850 (0.842-0.859)
MDRD eGFR (mL/min/1.73㎡)	2,076	92.8 (92.0-93.6)	2,294	89.0 (88.2-89.7)
CKD-EPI eGFR (mL/min/1.73㎡)	2,076	102.8 (102.0-103.6)	2,294	96.7 (95.9-97.5)
Random urine albumin (ug/mL)	2,076	23.7 (17.0-30.4)	2,294	19.6 (14.6-24.6)
Random urine creatinine (mg/dL)	2,076	152.0 (147.5-156.5)	2,294	159.2 (154.7-163.8)
Random urine ACR (mg/gCr)	2,076	29.7 (12.4-47.0)	2,294	19.8 (12.8-26.7)
CKD (Yes or No)	206	10.7 (9.2-12.3)	206	8.5 (7.3-9.8)
Fasting glucose (mg/dL)	2,076	95.2 (94.3-96.0)	2,294	98.6 (97.6-99.6)
HbA1c (%)	2,076	5.598 (5.568-5.629)	2,294	5.784 (5.748-5.821)

Variables	Meal frequency (n=4,370, weighted n=19,714,846)			
	MF <15		MF ≥15	
	n†	Mean or % (95% CI) ‡	n†	Mean or % (95% CI)‡
Total cholesterol (mg/dL)	2,076	186.9 (185.0-188.8)	2,294	188.4 (186.7-190.1)
HDL (mg/dL)	2,076	51.7 (51.1-52.3)	2,294	51.1 (50.5-51.6)
Triglyceride (mg/dL)	2,076	134.9 (128.8-141.1)	2,294	138.0 (132.3-143.6)
LDL (mg/dL)	2,076	111.4 (109.8-113.1)	2,294	112.5 (111.1-114.0)
Hemoglobin (g/dL)	2,076	14.36 (14.29-14.43)	2,294	14.36 (14.29-14.43)
Random urine sodium (mmol/L)	979	113.4 (109.2-117.5)	1,085	114.6 (110.7-118.6)
Vitamin D (ng/mL)	979	15.3 (14.8-15.8)	977	17.4 (16.8-18.0)
Daily Calorie intake (kcal/day)	2,076	2,195 (2,145-2,244)	2,294	2,199 (2,159-2,238)
Daily protein intake (g/day)	2,076	73.4 (71.4-75.5)	2,294	70.5 (69.0-72.0)
Daily fat intake (g/day)	2,076	48.6 (47.0-50.1)	2,294	40.9 (39.8-42.1)
Daily cholesterol intake (g/day)	2,076	291.5 (281.0-302.0)	2,294	249.4 (241.3-257.5)
Daily carbohydrate intake (g/day)	2,076	327.0 (320.5-333.5)	2,294	356.7 (351.0-362.4)
Daily calcium intake (mg/day)	2,076	522.8 (508.8-536.8)	2,294	510.6 (498.9-522.2)
Daily phosphorus intake (mg/day)	2,076	1,086 (1,062-1,115)	2,294	1,090 (1,069-1,111)
Daily sodium intake (mg/day)	2,076	3,661 (3,553-3,770)	2,294	3,489 (3,405-3,573)
Daily potassium intake (mg/day)	2,076	2,950 (2,874-3,027)	2,294	3,051 (2,987-3,116)
Daily sodium potassium intake ratio (n)	2,076	1.245 (1.229-1.262)	2,294	1.142 (1.127-1.157)
Body weight change				
no change	1,132	54.3 (51.6-57.0)	1,571	68.2 (65.8-70.4)
increase	658	31.3 (29.0-33.6)	428	18.3 (16.4-20.3)
decrease	285	14.4 (12.5-16.5)	295	13.5 (11.9-15.4)
Body weight control				
Maintain or not try	964	47.0 (44.8-49.3)	1,144	49.4 (47.1-51.7)
increase	111	6.1 (4.9-7.5)	134	6.7 (5.6-8.0)
decrease	1,001	46.9 (44.6-49.1)	1,016	43.9 (41.5-46.3)
Diet (Yes or No)	1,067	50.0 (47.8-52.3)	1,098	47.6 (45.3-50.0)
Diet by fasting	972	45.1 (42.8-47.5)	912	38.9 (36.6-41.3)
Diet for underlying disease	54	2.4 (1.7-3.2)	145	5.7 (4.7-6.9)

Variables	Meal frequency (n=4,370, weighted n=19,714,846)			
	MF <15		MF ≥15	
	n†	Mean or % (95% CI) ‡	n†	Mean or % (95% CI)‡
Body weight control				
Maintain or not try	964	47.0 (44.8-49.3)	1,144	49.4 (47.1-51.7)
increase	111	6.1 (4.9-7.5)	134	6.7 (5.6-8.0)
decrease	1,001	46.9 (44.6-49.1)	1,016	43.9 (41.5-46.3)
Diet (Yes or No)	1,067	50.0 (47.8-52.3)	1,098	47.6 (45.3-50.0)
Diet by fasting	972	45.1 (42.8-47.5)	912	38.9 (36.6-41.3)
Diet for underlying disease	54	2.4 (1.7-3.2)	145	5.7 (4.7-6.9)
Hypertension (Yes or No)	323	14.4 (12.7-16.4)	582	23.8 (21.8-25.9)
Diabetes (Yes or No)	123	5.1 (4.2-6.1)	280	11.0 (9.6-12.5)
Old Coronary arterial disease (Yes or No)	16	0.6 (0.3-1.0)	34	1.1 (0.8-1.6)
Myocardial infarction	5	0.2 (0.1-0.4)	9	0.3 (0.1-0.6)
Angina	12	0.4 (0.2-0.8)	28	0.9 (0.6-1.4)
Old cerebrovascular accident (Yes or No)	15	0.7 (0.4-1.3)	38	1.3 (0.8-2.0)
Dyslipidemia, (Yes or No)	685	33.2 (31.0-35.5)	880	37.4 (35.1-39.7)
Malignancy (Yes or No)	32	1.2 (0.8-1.9)	81	2.8 (2.1-3.7)
Depression (Yes or No)	155	6.9 (5.8-8.3)	120	4.9 (4.0-5.9)
PHQ-9 score (n)	977	3.20 (2.93-3.47)	1,080	2.15 (1.93-2.37)
Arthritis (Yes or No)	100	3.4 (2.7-4.3)	241	8.8 (7.7-10.1)
Osteoarthritis	82	2.7 (2.1-3.4)	208	7.5 (6.5-8.7)
Rheumatoid arthritis	20	0.8 (0.5-1.3)	36	1.4 (1.0-2.1)
Tuberculosis (Yes or No)	60	3.0 (2.2-4.0)	97	4.3 (3.4-5.4)
COPD (Yes or No)	47	2.2 (1.6-3.0)	147	5.6 (4.7-6.7)
Allergy (Yes or No)	407	20.1 (18.2-22.1)	395	18.2 (16.3-20.3)
Asthma	51	2.6 (1.9-3.5)	61	2.4 (1.8-3.2)
Atopic dermatitis	86	4.2 (3.3-5.3)	46	2.5 (1.9-3.4)
Rhinitis	326	16.0 (14.2-17.9)	324	14.9 (13.2-16.7)
Thyroid disease (Yes or No)	72	2.6 (2.0-3.4)	119	4.4 (3.6-5.3)

Variables	Meal frequency (n=4,370, weighted n=19,714,846)			
	MF <15		MF ≥15	
	n†	Mean or % (95% CI) ‡	n†	Mean or % (95% CI)‡
Viral liver disease (Yes or No)	86	4.4 (3.4-5.6)	120	5.0 (4.0-6.1)
Hepatitis B	81	4.1 (3.2-5.3)	106	4.4 (3.5-5.5)
Hepatitis C	5	0.3 (0.1-0.7)	7	0.3 (0.1-0.6)
Liver cirrhosis	0	0.0 (0.0-0.0)	12	0.5 (0.3-1.0)
2013 (enrolled year)	1,097	50.8 (44.6-57.0)	1,209	51.3 (45.3-57.3)
2014	979	49.2 (43.0-55.4)	1,085	48.7 (42.7-54.7)

Abbreviation: CI, confidence interval; NHIS, National Health Insurance Service; BP, blood pressure; BMI, body mass index; MET, metabolic equivalent task; MDRD, Modification of Diet in Renal Disease study; eGFR, estimated glomerular filtration rate; CKD-EPI, Chronic Kidney Disease-Epidemiology Collaboration; ACR, albumin creatinine ratio; CKD, chronic kidney disease; HbA1c, glycated hemoglobin; HDL, high density lipoprotein; LDL, low density lipoprotein; PHQ-9, Patient Health Questionnaire; COPD, chronic obstructive pulmonary disease.

MF <15: meal frequency <15times/week at least (n = 2,076, weighted n = 10,113,047.5).

MF ≥15: meal frequency ≥15times/week at least (n = 2,294, weighted n = 9,601,798.5).

†: unweighted n.

‡: weighted mean value or percentage with 95% confidence interval by complex samples analysis.

95 **Supplementary Table S2.** Baseline characteristics of the study population by breakfast frequency.

Variables	Breakfast frequency (n=4,370, weighted n=19,714,846)							
	seldom		1~2times/week		3~4times/week		5~7times/week	
	n†	Mean or % (95% CI) ‡	n†	Mean or % (95% CI)‡	n†	Mean or % (95% CI) ‡	n†	Mean or % (95% CI)‡
Male sex (Yes or No)	280	58.8 (54.5-62.9)	215	52.0 (47.3-56.6)	219	50.3 (45.8-54.9)	1,090	51.3 (49.3-53.3)
Age (years)	598	36.1 (35.0-37.2)	532	35.9 (34.9-36.9)	550	36.8 (35.7-38.0)	2,690	45.0 (44.4-45.6)
Annual family income								
High	175	30.4 (25.8-35.5)	193	37.5 (32.6-42.8)	173	31.3 (26.7-36.3)	929	35.1 (32.0-38.4)
Medium high	187	30.1 (25.8-34.9)	173	32.7 (28.1-37.7)	181	31.9 (27.5-36.7)	830	31.8 (29.1-34.5)
Medium low	176	29.7 (25.3-34.6)	128	23.4 (19.2-28.3)	155	29.9 (25.4-34.9)	664	24.2 (21.7-26.8)
Low	57	9.1 (6.6-12.4)	37	6.2 (4.4-8.7)	40	6.7 (4.6-9.7)	262	8.7 (7.4-10.3)
Education								
More than college	273	46.5 (42.1-50.9)	251	46.3 (41.0-51.8)	252	47.1 (42.1-52.1)	961	38.3 (35.8-40.9)
High school	236	41.6 (37.2-46.2)	223	44.1 (39.2-49.2)	236	43.8 (38.9-48.8)	1,029	40.7 (38.4-43.0)
Middle school	44	6.6 (4.6-9.4)	30	5.6 (3.8-8.1)	34	5.3 (3.7-7.6)	313	10.4 (9.1-12.0)
Less than elementary school	44	5.1 (3.5-7.4)	27	3.8 (2.4-5.8)	28	3.8 (2.5-5.8)	385	10.5 (9.2-12.0)
Job status (Yes or No)	404	68.9 (65.0-72.6)	366	72.2 (68.0-76.1)	359	66.2 (61.7-70.5)	1,755	68.0 (65.9-70.0)
Marital status								
married	367	55.9 (51.0-60.8)	353	60.2 (55.3-64.9)	370	60.4 (55.2-65.4)	2,176	76.7 (74.4-78.8)
divorce or widowed	42	5.1 (3.5-7.4)	33	4.8 (3.3-6.9)	36	4.7 (3.2-6.8)	188	5.8 (4.8-7.0)
not married	189	39.0 (34.0-44.2)	145	34.7 (30.0-39.7)	142	34.6 (29.8-39.7)	323	17.4 (15.5-19.5)

Variables	Breakfast frequency (n=4,370, weighted n=19,714,846)							
	seldom		1~2times/week		3~4times/week		5~7times/week	
	n†	Mean or % (95% CI) ‡	n†	Mean or % (95% CI)‡	n†	Mean or % (95% CI) ‡	n†	Mean or % (95% CI)‡
Insurance								
NHIS	567	94.9 (92.0-96.8)	510	95.9 (93.3-97.5)	539	98.2 (96.7-99.1)	2,620	97.4 (96.3-98.1)
medical aid	25	3.8 (2.2-6.4)	14	2.2 (1.1-4.4)	9	1.3 (0.6-2.7)	56	2.1 (1.4-3.0)
Personal insurance (Yes or No)	504	84.6 (80.6-87.9)	469	85.6 (81.5-88.9)	486	88.6 (85.4-91.1)	2,320	86.6 (84.6-88.4)
Smoking								
never	297	42.1 (37.8-46.6)	311	54.1 (49.1-58.9)	354	59.2 (54.5-63.8)	1,762	59.4 (57.3-61.3)
exsmoker	87	16.6 (13.3-20.5)	84	15.3 (12.2-19.1)	73	15.2 (12.1-18.8)	472	19.6 (17.9-21.4)
current	214	41.3 (37.0-45.8)	137	30.6 (26.2-35.4)	123	25.6 (21.7-30.0)	456	21.1 (19.0-23.3)
Alcohol consumption, ever	567	95.1 (92.7-96.8)	502	95.7 (93.6-97.1)	517	94.3 (91.9-96.0)	2,431	92.4 (91.2-93.4)
Alcohol consumption in last 1 year	376	67.2 (63.0-71.2)	363	70.4 (65.8-74.7)	348	65.7 (61.1-69.9)	1,405	57.0 (54.8-59.3)
EuroQol-5D (n)	598	0.963 (0.955-0.970)	532	0.971 (0.965-0.976)	550	0.968 (0.962-0.974)	2,690	0.964 (0.959-0.968)
Systolic BP (mmHg)	597	113.1 (111.9-114.2)	532	113.3 (111.9-114.8)	550	113.3 (111.9-114.6)	2,689	115.7 (114.9-116.4)
Diastolic BP (mmHg)	597	75.1 (74.2-76.0)	532	75.2 (74.2-76.3)	550	75.4 (74.4-76.5)	2,689	76.1 (75.6-76.6)
Waist circumference (cm)	598	80.0 (79.0-81.0)	532	79.7 (78.8-80.7)	550	79.7 (78.8-80.7)	2,690	80.7 (80.3-81.2)
Abdominal obesity (Yes or No)	76	15.9 (12.5-20.0)	50	12.3 (9.4-16.0)	56	12.8 (9.8-16.4)	306	13.8 (12.3-15.5)
BMI (kg/㎡)	598	23.4 (23.1-23.8)	532	23.8 (23.4-24.1)	550	23.6 (23.2-23.9)	2,690	23.8 (23.7-24.0)

Variables	Breakfast frequency (n=4,370, weighted n=19,714,846)							
	seldom		1~2times/week		3~4times/week		5~7times/week	
	n†	Mean or % (95% CI) ‡	n†	Mean or % (95% CI)‡	n†	Mean or % (95% CI) ‡	n†	Mean or % (95% CI)‡
Obesity in BMI								
Normal to pre-obesity (18.5~25)	394	65.3 (60.4-69.9)	328	59.0 (54.3-63.6)	361	63.4 (58.6-68.0)	1,720	64.1 (62.0-66.2)
Normal (18.5~23)	266	43.6 (39.0-48.3)	219	40.5 (35.9-45.2)	237	40.1 (35.3-45.0)	1,051	38.8 (36.7-40.8)
Pre-obesity (23~25)	128	21.7 (18.1-25.7)	109	18.6 (15.5-22.1)	124	23.3 (19.5-27.7)	669	25.4 (23.6-27.2)
Obesity (≥25)	170	29.1 (24.7-33.9)	173	35.6 (31.2-40.2)	164	31.4 (27.2-36.0)	877	32.5 (30.5-34.6)
1st obesity (25~30)	143	24.3 (20.4-28.6)	147	29.6 (25.3-34.4)	140	26.8 (22.7-31.4)	767	28.1 (26.3-30.0)
2nd obesity (30~35)	25	4.6 (3.0-6.8)	22	4.9 (3.1-7.8)	21	4.3 (2.8-6.6)	100	4.1 (3.2-5.2)
3rd obesity (≥35)	2	0.3 (0.1-1.3)	4	1.0 (0.3-2.8)	3	0.3 (0.1-1.1)	10	0.3 (0.1-0.6)
Under weighted (<18.5)	34	5.6 (3.8-8.1)	31	5.4 (3.7-7.7)	25	5.2 (3.4-7.8)	93	3.3 (2.6-4.2)
Exercise (Yes or No)	229	42.4 (37.6-47.3)	249	50.3 (45.2-55.3)	268	50.6 (45.4-55.7)	1,256	49.4 (47.0-51.8)
More than 150 mins/week	163	30.9 (26.5-35.7)	181	37.4 (33.0-42.1)	196	37.4 (32.3-42.7)	933	37.0 (34.8-39.4)
Physical activity (MET-minutes/week)	598	1,975 (1,616-2,334)	532	1,844 (1,630-2,059)	550	1,820 (1,586-2,054)	2,690	1,847 (1,719-1,974)
High (≥3000)	97	18.5 (15.0-22.6)	96	20.9 (17.0-25.3)	89	16.4 (13.1-20.3)	463	18.7 (17.0-20.5)
Moderate (600-3000)	224	38.3 (34.1-42.7)	235	44.5 (39.6-49.6)	264	50.1 (45.2-55.0)	1,152	43.4 (41.3-45.6)
Low (<600)	277	43.2 (38.7-47.7)	201	34.6 (30.2-39.3)	197	33.5 (28.9-38.5)	1,075	37.9 (35.6-40.2)
Serum creatinine (mg/dL)	598	0.865 (0.848-0.882)	532	0.843 (0.828-0.857)	550	0.842 (0.824-0.859)	2,690	0.848 (0.837-0.860)
MDRD eGFR (mL/min/1.73㎡)	598	92.8 (91.4-94.1)	532	93.1 (91.8-94.4)	550	93.3 (91.9-94.7)	2,690	89.3 (88.6-90.0)
CKD-EPI eGFR (mL/min/1.73㎡)	598	103.0 (101.7-104.3)	532	103.8 (102.6-105.1)	550	103.3 (101.9-104.7)	2,690	97.2 (96.5-97.9)

**Variables**	**Breakfast frequency (n=4,370, weighted n=19,714,846)**							
	**seldom**		**1~2times/week**		**3~4times/week**		**5~7times/week**	
	n†	Mean or % (95% CI) ‡	n†	Mean or % (95% CI)‡	n†	Mean or % (95% CI) ‡	n†	Mean or % (95% CI)‡
Random urine albumin (ug/mL)	598	30.4 (18.3-42.6)	532	15.5 (12.0-19.1)	550	14.8 (10.9-18.7)	2,690	22.3 (16.0-28.6)
Random urine creatinine (mg/dL)	598	153.5 (144.8-162.2)	532	155.5 (146.2-164.9)	550	149.1 (141.1-157.2)	2,690	157.6 (153.5-161.8)
Random urine ACR (mg/gCr)	598	34.2 (17.0-51.3)	532	14.1 (11.1-17.2)	550	14.4 (9.6-19.2)	2,690	27.3 (11.5-43.0)
CKD (Yes or No)	66	11.6 (9.0-14.9)	55	10.5 (8.0-13.8)	47	9.7 (7.3-12.7)	244	8.8 (7.7-10.1)
Fasting glucose (mg/dL)	598	95.3 (93.8-96.8)	532	94.6 (93.2-96.0)	550	94.6 (93.2-95.9)	2,690	98.3 (97.4-99.2)
HbA1c (%)	598	5.580 (5.528-5.631)	532	5.561 (5.507-5.615)	550	5.575 (5.529-5.622)	2,690	5.777 (5.742-5.811)
Total cholesterol (mg/dL)	598	188.0 (184.4-191.7)	532	189.6 (186.1-193.0)	550	182.1 (179.3-185.0)	2,690	188.4 (186.8-189.9)
HDL (mg/dL)	598	51.7 (50.6-52.8)	532	51.5 (50.3-52.6)	550	51.8 (50.8-52.7)	2,690	51.2 (50.6-51.7)
Triglyceride (mg/dL)	598	141.9 (128.7-155.2)	532	140.7 (128.4-153.1)	550	122.4 (113.5-131.3)	2,690	137.2 (132.2-142.2)
LDL (mg/dL)	598	110.9 (108.1-113.8)	532	114.0 (110.8-117.2)	550	108.4 (105.8-111.0)	2,690	112.6 (111.3-113.9)
Hemoglobin (g/dL)	598	14.60 (14.47-14.74)	532	14.36 (14.20-14.52)	550	14.31 (14.18-14.45)	2,690	14.30 (14.23-14.37)
Random urine sodium (mmol/L)	295	111.8 (105.1-118.4)	235	118.0 (111.0-124.9)	265	111.2 (104.1-118.2)	1,269	114.4 (111.9-117.9)
Vitamin D (ng/mL)	297	14.5 (13.9-15.2)	267	15.3 (14.4-16.2)	241	15.9 (15.0-16.8)	1,151	17.2 (16.7-17.7)
Daily Calorie intake (kcal/day)	598	2,126 (2,050-2,203)	532	2,246 (2,151-2,341)	550	2,342 (2,246-2,439)	2,690	2,170 (2,133-2,207)
Daily protein intake (g/day)	598	70.1 (67.1-73.1)	532	76.0 (72.2-79.8)	550	79.2 (75.1-83.4)	2,690	69.9 (68.5-71.3)
Daily fat intake (g/day)	598	47.1 (44.7-49.5)	532	51.3 (48.3-54.2)	550	51.7 (48.6-54.8)	2,690	41.1 (40.0-42.1)
Daily cholesterol intake (g/day)	598	285.5 (270.4-300.6)	532	302.5 (281.0-324.0)	550	310.1 (289.8-330.4)	2,690	250.2(242.8-257.7)
Daily carbohydrate intake (g/day)	598	313.3 (302.8-323.8)	532	325.9 (313.4-338.5)	550	354.6 (342.0-367.3)	2,690	349.8 (344.5-355.1)

Variables	Breakfast frequency (n=4,370, weighted n=19,714,846)							
	seldom		1~2times/week		3~4times/week		5~7times/week	
	n†	Mean or % (95% CI) ‡	n†	Mean or % (95% CI)‡	n†	Mean or % (95% CI) ‡	n†	Mean or % (95% CI)‡
Daily calcium intake (mg/day)	598	492.9 (471.3-514.6)	532	543.9 (516.6-571.1)	550	548.6 (522.3-574.9)	2,690	509.6 (498.5-520.8)
Daily phosphorus intake (mg/day)	598	1,031 (982-1,071)	532	1,114 (1,061-1,167)	550	1,164 (1,112-1,217)	2,690	1,081 (1,061-1,101)
Daily sodium intake (mg/day)	598	3,512 (3,347-3,677)	532	3,830 (3,621-4,038)	550	3,880 (3,671-4,088)	2,690	3,464 (3,385-3,543)
Daily potassium intake (mg/day)	598	2,738 (2,614-2,861)	532	3,036 (2,886-3,186)	550	3,138 (2,994-3,283)	2,690	3,031 (2,970-3,092)
Daily sodium potassium intake ratio (n)	598	1.295 (1.264-1.327)	532	1.269 (1.241-1.297)	550	1.224 (1.197-1.252)	2,690	1.143 (1.128-1.157)
Body weight change								
no change	325	54.0 (49.6-58.3)	280	53.4 (48.4-58.4)	319	55.8 (50.9-60.7)	1,779	66.1 (63.8-68.2)
increase	196	32.7 (28.7-37.0)	179	32.6 (28.0-37.4)	151	28.6 (24.4-33.3)	560	20.1 (18.3-22.1)
decrease	76	13.3 (10.3-17.0)	73	14.0 (11.0-17.7)	80	15.6 (12.2-19.6)	351	13.8 (12.2-15.5)
Body weight control								
Maintain or not try	296	50.7 (46.2-55.2)	258	48.8 (44.3-53.3)	251	45.3 (40.8-49.9)	1,303	48.1 (45.9-50.3)
increase	38	7.2 (5.1-10.1)	36	7.6 (5.5-10.4)	25	5.2 (3.3-7.9)	146	6.2 (5.2-7.3)
decrease	264	42.1 (37.6-46.8)	238	43.6 (39.1-48.2)	274	49.5 (45.0-54.0)	1,241	45.8 (43.6-48.0)
Diet (Yes or No)	276	44.0 (39.5-48.6)	257	47.5 (43.0-52.0)	287	52.3 (47.8-56.8)	1,345	49.7 (47.5-51.9)
Diet by fasting	252	39.0 (35.0-43.2)	231	43.5 (39.0-48.0)	260	45.8 (41.2-50.5)	1,141	41.8 (39.6-44.0)
Diet for underlying disease	13	1.9 (1.0-3.7)	11	1.6 (0.9-2.9)	18	3.1 (1.9-5.0)	157	5.3 (4.4-6.4)
Hypertension (Yes or No)	88	13.8 (11.1-17.0)	76	13.6 (10.6-17.4)	78	13.0 (10.4-16.2)	663	23.2 (21.3-25.1)
Diabetes (Yes or No)	35	5.2 (3.6-7.3)	19	3.5 (2.1-5.8)	33	4.7 (3.2-6.9)	316	10.5 (9.3-11.9)

Variables	Breakfast frequency (n=4,370, weighted n=19,714,846)							
	seldom		1~2times/week		3~4times/week		5~7times/week	
	n†	Mean or % (95% CI) ‡	n†	Mean or % (95% CI)‡	n†	Mean or % (95% CI) ‡	n†	Mean or % (95% CI)‡
Old Coronary arterial disease (Yes or No)	7	1.0 (0.5-2.3)	0	0.0 (0.0-0.0)	2	0.3 (0.1-1.1)	41	1.1 (0.8-1.6)
Myocardial infarction	2	0.3 (0.1-1.3)	0	0.0 (0.0-0.0)	0	0.0 (0.0-0.0)	12	0.3 (0.2-0.5)
Angina	5	0.7 (0.3-1.9)	0	0.0 (0.0-0.0)	2	0.3 (0.1-1.1)	33	0.9 (0.6-1.4)
Old cerebrovascular accident (Yes or No)	6	0.8 (0.3-2.1)	3	0.8 (0.3-2.8)	2	0.4 (0.1-1.5)	42	1.2 (0.8-1.9)
Dyslipidemia, (Yes or No)	204	33.6 (29.6-37.8)	182	37.1 (32.8-41.6)	153	27.4 (23.6-31.5)	1,026	37.2 (35.1-39.3)
Malignancy (Yes or No)	5	0.7 (0.3-1.9)	4	0.5 (0.2-1.4)	10	1.4 (0.8-2.7)	94	2.9 (2.2-3.7)
Depression (Yes or No)	60	8.9 (6.6-11.7)	37	7.3 (5.0-10.7)	25	4.0 (2.5-6.1)	153	5.3 (4.4-6.3)
PHQ-9 score (n)	293	3.67 (3.09-4.26)	235	3.31 (2.79-3.82)	265	2.48 (2.07-2.88)	1,264	2.32 (2.12-2.53)
Arthritis (Yes or No)	21	2.9 (1.7-4.8)	23	2.9 (1.8-4.5)	27	3.5 (2.2-5.4)	270	8.3 (7.2-9.5)
Osteoarthritis	16	2.2 (1.2-4.0)	19	2.5 (1.6-4.1)	22	2.3 (1.4-3.8)	233	7.0 (6.1-8.1)
Rheumatoid arthritis	5	0.6 (0.2-1.7)	5	0.4 (0.1-1.3)	6	1.2 (0.5-2.9)	40	1.4 (1.0-1.9)
Tuberculosis (Yes or No)	19	2.8 (1.6-4.7)	19	3.9 (2.4-6.3)	11	2.4 (1.3-4.4)	108	4.1 (3.2-5.1)
COPD (Yes or No)	18	2.8 (1.7-4.5)	7	1.5 (0.7-3.4)	9	1.7 (0.8-3.5)	160	5.2 (4.4-6.2)
Allergy (Yes or No)	118	19.4 (16.4-22.9)	109	21.1 (17.4-25.4)	111	20.9 (17.2-25.2)	464	18.2 (16.4-20.1)
Asthma	13	2.4 (1.3-4.2)	11	2.5 (1.1-5.4)	18	3.3 (1.9-5.4)	70	2.4 (1.8-3.1)
Atopic dermatitis	31	5.0 (3.4-7.2)	22	4.9 (3.1-7.5)	25	4.4 (2.9-6.7)	54	2.3 (1.7-3.1)
Rhinitis	91	14.6 (11.7-18.0)	88	16.3 (13.2-20.0)	88	17.0 (13.5-21.2)	383	15.1 (13.5-16.8)
Thyroid disease (Yes or No)	14	1.7 (0.9-3.4)	13	1.9 (1.1-3.3)	20	2.9 (1.8-4.7)	144	4.4 (3.7-5.3)

**Variables**	**Breakfast frequency (n=4,370, weighted n=19,714,846)**							
	**seldom**		**1~2times/week**		**3~4times/week**		**5~7times/week**	
	n†	Mean or % (95% CI) ‡	n†	Mean or % (95% CI)‡	n†	Mean or % (95% CI) ‡	n†	Mean or % (95% CI)‡
Viral liver disease (Yes or No)	29	5.2 (3.5-7.7)	19	3.1 (1.9-4.9)	23	4.6 (2.7-7.6)	135	4.9 (4.0-6.0)
Hepatitis B	28	5.1 (3.4-7.6)	19	3.1 (1.9-4.9)	21	4.2 (2.4-7.1)	119	4.3 (3.5-5.3)
Hepatitis C	1	0.1 (0.0-0.5)	0	0.0 (0.0-0.0)	2	0.4 (0.1-2.0)	9	0.3 (0.2-0.7)
Liver cirrhosis	0	0.0 (0.0-0.0)	0	0.0 (0.0-0.0)	0	0.0 (0.0-0.0)	12	0.4 (0.2-0.8)
2013 (enrolled year)	303	51.0 (43.4-58.5)	297	51.3 (43.9-58.7)	285	49.3 (41.7-57.0)	1,421	51.4 (45.5-57.3)
2014	295	49.0 (41.5-56.6)	235	48.7 (41.3-56.1)	265	50.7 (43.0-58.3)	1,269	48.6 (42.7-54.5)

Seldom : n = 598, weighted n = 3,143,377.
1~2times/week : n = 532, weighted n = 2,625,784.
3~4times/week : n = 550, weighted n = 2,694,635.
5~7times/week : n = 2,690, weighted n = 11,251,050.

Supplementary Table S3. Baseline characteristics of the study population by lunch frequency.

Variables	Lunch frequency (n=4,370, weighted n=19,714,846)							
	seldom		1~2times/week		3~4times/week		5~7times/week	
	n†	Mean or % (95% CI) ‡	n†	Mean or % (95% CI)‡	n†	Mean or % (95% CI) ‡	n†	Mean or % (95% CI)‡
Male sex (Yes or No)	17	42.4 (28.2-58.0)	30	41.8 (30.6-53.9)	100	42.0 (35.9-48.2)	1,657	53.8 (52.1-55.5)
Age (years)	64	40.5 (36.2-44.7)	85	37.5 (34.2-40.8)	323	37.4 (36.0-38.9)	3,898	41.7 (41.1-42.3)
Annual family income								
High	13	21.3 (11.4-36.3)	25	29.7 (19.8-42.0)	85	26.8 (21.7-32.5)	1,347	35.2 (32.4-38.0)
Medium high	19	27.8 (17.2-41.8)	25	30.0 (20.0-42.3)	109	33.7 (28.0-39.8)	1,218	31.6 (29.4-33.9)
Medium low	22	35.5 (22.5-51.1)	26	28.9 (19.2-41.0)	92	29.9 (24.2-36.3)	983	25.1 (22.9-27.5)
Low	10	15.3 (7.0-30.4)	9	11.4 (5.7-21.6)	36	9.2 (6.2-13.3)	341	7.9 (6.8-9.2)
Education								
More than college	20	32.7 (21.0-47.1)	27	35.8 (24.9-48.4)	129	40.6 (34.4-47.2)	1,561	42.3 (40.1-44.5)
High school	27	48.4 (34.1-63.0)	38	43.0 (31.4-55.3)	132	43.6 (37.4-50.1)	1,527	41.4 (39.4-43.4)
Middle school	3	3.5 (1.0-11.3)	8	9.1 (4.4-17.8)	35	9.3 (6.4-13.2)	375	8.5 (7.4-9.7)
Less than elementary school	14	15.4 (8.4-26.4)	12	12.2 (6.3-22.3)	27	6.5 (4.2-9.8)	431	7.7 (6.8-8.8)
Job status (Yes or No)	34	52.9 (38.4-67.0)	46	59.7 (47.7-70.6)	173	57.9 (51.9-63.7)	2,631	69.8 (68.1-71.5)
Marital status								
married	37	52.1 (38.1-65.7)	55	55.4 (43.6-66.7)	226	63.8 (57.2-69.9)	2,948	70.0 (67.8-72.2)
divorce or widowed	11	10.1 (5.1-19.3)	7	6.4 (2.8-14.1)	22	5.9 (3.2-10.5)	259	5.3 (4.5-6.1)
not married	16	37.8 (24.7-53.0)	23	38.2 (26.9-50.9)	75	30.4 (24.6-36.8)	685	24.6 (22.5-26.8)

**Variables**	**Lunch frequency (n=4,370, weighted n=19,714,846)**							
	**seldom**		**1~2times/week**		**3~4times/week**		**5~7times/week**	
	n†	Mean or % (95% CI) ‡	n†	Mean or % (95% CI)‡	n†	Mean or % (95% CI) ‡	n†	Mean or % (95% CI)‡
Insurance								
NHIS	58	92.3 (82.6-96.8)	77	89.5 (77.6-95.4)	307	94.8 (89.9-97.4)	3,794	97.3 (96.5-98.0)
medical aid	5	7.3 (2.9-17.1)	7	8.2 (3.2-19.2)	12	3.8 (1.9-7.4)	80	1.9 (1.3-2.7)
Personal insurance (Yes or No)	50	80.8 (67.6-89.4)	72	85.7 (75.0-92.3)	275	84.4 (79.0-88.6)	3,382	86.7 (85.1-88.1)
Smoking								
never	39	50.2 (35.8-64.5)	45	46.9 (35.4-58.7)	205	56.6 (50.8-62.3)	2,435	56.1 (54.3-57.9)
exsmoker	7	13.8 (6.1-28.3)	8	8.6 (3.8-18.1)	35	11.4 (8.1-15.7)	666	18.8 (17.4-20.2)
current	18	36.1 (23.0-51.6)	32	44.5 (33.0-56.6)	83	32.0 (26.4-38.1)	797	25.1 (23.3-27.0)
Alcohol consumption, ever	59	94.4 (85.9-97.9)	79	89.7 (77.3-95.7)	301	94.5 (91.3-96.6)	3,578	93.5 (92.5-94.4)
Alcohol consumption in last 1 year	32	58.0 (43.7-71.1)	55	67.8 (56.2-77.5)	190	62.4 (56.5-68.1)	2,215	61.5 (59.5-63.4)
EuroQol-5D (n)	64	0.957 (0.939-0.974)	85	0.941 (0.915-0.968)	323	0.956 (0.946-0.967)	3,898	0.966 (0.963-0.969)
Systolic BP (mmHg)	64	117.6 (112.5-122.6)	84	115.1 (111.7-118.4)	322	112.1 (110.2-114.1)	3,898	114.8 (114.2-115.4)
Diastolic BP (mmHg)	64	76.2 (73.3-79.1)	84	74.8 (72.3-77.4)	322	74.3 (72.9-75.8)	3,898	75.9 (75.4-76.3)
Waist circumference (cm)	64	80.8 (78.0-83.6)	85	80.1 (77.7-82.4)	323	79.1 (77.9-80.3)	3,898	80.5 (80.1-80.8)
Abdominal obesity (Yes or No)	5	14.7 (6.0-31.8)	13	16.2 (9.3-26.8)	19	7.5 (4.6-12.1)	451	14.3 (13.0-15.7)
BMI (kg/㎡)	64	23.7 (22.7-24.6)	85	24.0 (23.0-25.0)	323	23.6 (23.1-24.0)	3,898	23.7 (23.6-23.9)

Variables	Lunch frequency (n=4,370, weighted n=19,714,846)							
	seldom		1~2times/week		3~4times/week		5~7times/week	
	n†	Mean or % (95% CI) ‡	n†	Mean or % (95% CI)‡	n†	Mean or % (95% CI) ‡	n†	Mean or % (95% CI)‡
Obesity in BMI								
Normal to pre-obesity (18.5~25)	37	54.6 (40.3-68.2)	47	55.8 (44.2-66.8)	217	66.2 (59.9-71.9)	2,502	63.6 (61.8-65.4)
Normal (18.5~23)	28	40.3 (27.5-54.5)	33	37.8 (27.0-49.9)	136	41.2 (35.5-47.2)	1,576	39.9 (38.1-41.6)
Pre-obesity (23~25)	9	14.3 (7.1-26.8)	14	18.0 (10.4-29.4)	81	25.0 (20.4-30.2)	926	23.7 (22.3-25.3)
Obesity (≥25)	23	39.3 (26.0-54.5)	34	38.3 (28.3-49.4)	89	28.2 (22.8-34.3)	1,238	32.3 (30.6-34.2)
1st obesity (25~30)	20	34.3 (21.5-49.7)	29	32.2 (22.7-43.4)	72	23.8 (18.7-29.7)	1,076	27.7 (26.0-29.3)
2nd obesity (30~35)	3	5.1 (1.4-16.9)	5	6.1 (2.5-14.5)	14	3.4 (1.9-6.0)	146	4.3 (3.6-5.2)
3rd obesity (≥35)	0	0.0 (0.0-0.0)	0	0.0 (0.0-0.0)	3	1.1 (0.3-3.4)	16	0.4 (0.2-0.6)
Under weighted (<18.5)	4	6.1 (2.2-15.8)	4	5.9 (2.0-15.9)	17	5.6 (3.2-9.5)	158	4.0 (3.4-4.8)
Exercise (Yes or No)	22	41.5 (27.9-56.6)	34	41.7 (30.5-53.8)	134	43.2 (37.1-49.6)	1,812	49.3 (47.1-51.5)
More than 150 mins/week	18	35.0 (21.8-51.0)	29	36.4 (25.8-48.6)	100	31.1 (25.6-37.1)	1,326	36.6 (34.7-38.6)
Physical activity (MET-minutes/week)	64	1,851 (1,230-2,473)	85	1,397 (982-1,813)	323	2,152 (1,677-2,628)	3,898	1,848 (1,737-1,958)
High (≥3000)	11	19.6 (10.6-33.4)	11	14.3 (7.6-25.5)	66	22.6 (17.6-28.4)	657	18.4 (16.9-19.9)
Moderate (600-3000)	18	34.3 (21.8-49.6)	32	36.5 (26.0-48.6)	131	39.8 (34.2-45.6)	1,694	44.3 (42.5-46.2)
Low (<600)	35	46.0 (32.8-59.8)	42	49.1 (37.8-60.5)	126	37.7 (32.0-43.7)	1,547	37.3 (35.4-39.3)
Serum creatinine (mg/dL)	64	0.823 (0.776-0.870)	85	0.800 (0.767-0.833)	323	0.853 (0.784-0.923)	3,898	0.851 (0.844-0.857)
MDRD eGFR (mL/min/1.73㎡)	64	91.9 (87.5-96.4)	85	94.9 (92.3-97.5)	323	92.3 (90.4-94.3)	3,898	90.7 (90.1-91.3)
CKD-EPI eGFR (mL/min/1.73㎡)	64	101.2 (96.7-105.8)	85	105.6 (102.7-108.6)	323	102.6 (100.7-104.5)	3,898	99.4 (98.8-100.1)

Variables	Lunch frequency (n=4,370, weighted n=19,714,846)							
	seldom		1~2times/week		3~4times/week		5~7times/week	
	n†	Mean or % (95% CI) ‡	n†	Mean or % (95% CI)‡	n†	Mean or % (95% CI) ‡	n†	Mean or % (95% CI)‡
Random urine albumin (ug/mL)	64	18.8 (5.8-31.9)	85	12.7 (7.9-17.5)	323	32.1 (-0.9-65.2)	3,898	21.0 (17.2-24.7)
Random urine creatinine (mg/dL)	64	150.0 (122.0-178.0)	85	151.7 (129.5-173.8)	323	154.4 (143.4-165.5)	3,898	155.8 (152.2-159.4)
Random urine ACR (mg/gCr)	64	28.9 (-2.1-60.0)	85	12.9 (8.7-17.0)	323	69.6 (-36.1-175.4)	3,898	21.0 (16.1-26.0)
CKD (Yes or No)	6	13.7 (5.8-29.2)	7	10.7 (5.0-21.2)	34	11.3 (7.9-15.9)	365	9.4 (8.3-10.5)
Fasting glucose (mg/dL)	64	95.3 (91.7-98.9)	85	96.4 (92.9-99.9)	323	95.2 (92.8-97.6)	3,898	97.0 (96.3-97.7)
HbA1c (%)	64	5.647 (5.492-5.802)	85	5.625 (5.490-5.760)	323	5.593 (5.527-5.658)	3,898	5.700 (5.672-5.727)
Total cholesterol (mg/dL)	64	187.4 (178.7-196.1)	85	187.6 (178.8-196.5)	323	186.8 (182.2-191.4)	3,898	187.7 (186.4-189.0)
HDL (mg/dL)	64	49.3 (46.1-52.5)	85	51.4 (48.9-53.9)	323	52.5 (51.3-53.8)	3,898	51.3 (50.9-51.7)
Triglyceride (mg/dL)	64	176.3 (131.9-220.7)	85	135.7 (110.6-160.8)	323	127.4 (111.7-143.1)	3,898	136.6 (132.1-141.1)
LDL (mg/dL)	64	109.3 (102.1-116.5)	85	113.1 (105.1-121.1)	323	112.4 (108.3-116.4)	3,898	111.9 (110.8-113.1)
Hemoglobin (g/dL)	64	14.40 (13.81-14.99)	85	14.19 (13.83-14.55)	323	14.08 (13.89-14.28)	3,898	14.39 (14.33-14.44)
Random urine sodium (mmol/L)	37	117.0 (91.5-142.5)	42	111.9 (95.3-128.4)	167	105.0 (95.4-114.7)	1,818	114.9 (112.0-117.8)
Vitamin D (ng/mL)	26	14.5 (12.3-16.7)	42	14.9 (13.1-16.8)	145	14.7 (13.3-16.1)	1,743	16.5 (16.0-16.9)
Daily Calorie intake (kcal/day)	64	1,747 (1,561-1,932)	85	1,864 (1,662-2,065)	323	2,194 (2,062-2,325)	3,898	2,211 (2,179-2,244)
Daily protein intake (g/day)	64	56.7 (48.1-65.4)	85	59.5 (51.9-67.0)	323	73.8 (68.1-79.6)	3,898	72.4 (71.0-73.7)
Daily fat intake (g/day)	64	36.3 (29.7-43.0)	85	39.9 (34.1-45.6)	323	49.1 (44.8-53.5)	3,898	44.7 (43.7-45.7)
Daily cholesterol intake (g/day)	64	240.6 (187.6-293.5)	85	253.0 (221.5-294.5)	323	302.5 (273.2-331.7)	3,898	269.1(262.1-276.1)
Daily carbohydrate intake (g/day)	64	273.6 (249.1-298.2)	85	267.9 (241.7-294.2)	323	330.0 (313.0-347.0)	3,898	345.2 (340.7-349.7)

**Variables**	**Lunch frequency (n=4,370, weighted n=19,714,846)**							
	**seldom**		**1~2times/week**		**3~4times/week**		**5~7times/week**	
	n†	Mean or % (95% CI) ‡	n†	Mean or % (95% CI)‡	n†	Mean or % (95% CI) ‡	n†	Mean or % (95% CI)‡
Daily calcium intake (mg/day)	64	406.7 (351.5-462.0)	85	440.3 (380.3-500.3)	323	537.0 (496.7-577.3)	3,898	518.5 (509.4-527.6)
Daily phosphorus intake (mg/day)	64	869 (757-982)	85	908 (799-1,016)	323	1,113 (1,035-1,190)	3,898	1,095 (1,077-1,112)
Daily sodium intake (mg/day)	64	2,751 (2,251-3,251)	85	2,933 (2,559-3,307)	323	3,682 (3,393-3,972)	3,898	3,596 (3,522-3,669)
Daily potassium intake (mg/day)	64	2,511 (2,168-2,853)	85	2,425 (2,147-2,703)	323	3,072 (2,846-3,298)	3,898	3,014 (2,962-3,065)
Daily sodium potassium intake ratio (n)	64	1.102 (0.994-1.210)	85	1.230 (1.138-1.322)	323	1.201 (1.165-1.236)	3,898	1.195 (1.182-1.208)
Body weight change								
no change	40	66.3 (52.7-77.6)	39	43.2 (31.7-55.5)	159	47.8 (41.5-54.1)	2,465	62.6 (60.6-64.4)
increase	14	22.2 (12.6-36.0)	28	32.6 (22.6-44.4)	121	37.9 (32.1-44.2)	923	23.7 (22.1-25.3)
decrease	10	11.6 (5.9-21.5)	18	24.2 (15.1-36.4)	43	14.3 (10.3-19.5)	509	13.8 (12.4-15.2)
Body weight control								
Maintain or not try	29	47.5 (33.6-61.8)	43	49.8 (37.3-62.3)	139	44.7 (38.4-51.2)	1,897	48.5 (46.8-50.2)
increase	2	1.5 (0.3-7.1)	3	5.2 (1.7-14.8)	18	6.5 (3.9-10.8)	222	6.5 (5.6-7.4)
decrease	33	50.9 (36.8-64.9)	39	45.0 (33.2-57.4)	166	48.7 (42.4-55.1)	1,779	45.0 (43.3-46.7)
Diet (Yes or No)	34	51.2 (37.0-65.2)	41	46.8 (34.9-59.2)	181	54.7 (48.2-61.0)	1,909	48.4 (46.7-50.0)
Diet by fasting	30	44.9 (31.3-59.3)	39	48.3 (36.7-60.2)	163	49.6 (43.4-55.7)	1,652	41.2 (39.5-43.0)
Diet for underlying disease	1	0.6 (0.1-4.2)	1	1.1 (0.2-7.6)	5	1.1 (0.4-2.8)	192	4.4 (3.7-5.2)
Hypertension (Yes or No)	13	17.2 (9.7-28.5)	8	6.7 (3.0-14.3)	47	12.7 (9.3-17.1)	837	19.9 (18.4-21.5)
Diabetes (Yes or No)	5	7.9 (3.0-19.1)	4	2.6 (0.8-7.4)	22	5.9 (3.8-8.9)	372	8.2 (7.3-9.3)

Variables	Lunch frequency (n=4,370, weighted n=19,714,846)							
	seldom		1~2times/week		3~4times/week		5~7times/week	
	n†	Mean or % (95% CI) ‡	n†	Mean or % (95% CI)‡	n†	Mean or % (95% CI) ‡	n†	Mean or % (95% CI)‡
Old Coronary arterial disease (Yes or No)	0	0.0 (0.0-0.0)	0	0.0 (0.0-0.0)	6	1.4 (0.6-3.5)	44	0.8 (0.6-1.1)
Myocardial infarction	0	0.0 (0.0-0.0)	0	0.0 (0.0-0.0)	3	0.6 (0.2-2.3)	11	0.2 (0.1-0.4)
Angina	0	0.0 (0.0-0.0)	0	0.0 (0.0-0.0)	3	0.8 (0.2-2.7)	37	0.7 (0.5-1.0)
Old cerebrovascular accident (Yes or No)	1	0.8 (0.1-5.7)	0	0.0 (0.0-0.0)	4	0.9 (0.3-2.7)	48	1.0 (0.7-1.5)
Dyslipidemia, (Yes or No)	31	48.7 (34.4-63.2)	30	34.3 (24.5-45.5)	95	29.8 (24.0-36.4)	1,409	35.5 (33.9-37.2)
Malignancy (Yes or No)	2	2.1 (0.3-13.3)	2	1.5 (0.4-5.9)	9	2.0 (1.0-3.8)	100	2.0 (1.6-2.6)
Depression (Yes or No)	11	19.1 (10.1-33.2)	8	7.6 (3.7-15.1)	32	8.7 (5.9-12.6)	224	5.4 (4.6-6.4)
PHQ-9 score (n)	37	5.13 (3.45-6.80)	42	4.41 (2.94-5.88)	167	3.50 (2.85-4.15)	1,811	2.52 (2.33-2.71)
Arthritis (Yes or No)	2	1.6 (0.4-6.6)	7	6.2 (2.7-13.4)	19	4.2 (2.4-7.2)	313	6.3 (5.6-7.1)
Osteoarthritis	1	0.9 (0.1-6.5)	6	5.6 (2.3-12.7)	16	3.2 (1.8-5.8)	267	5.2 (4.6-6.0)
Rheumatoid arthritis	1	0.7 (0.1-4.9)	1	0.6 (0.1-4.3)	4	1.1 (0.3-3.6)	50	1.1 (0.8-1.5)
Tuberculosis (Yes or No)	4	4.6 (1.5-13.2)	4	5.4 (1.7-15.7)	7	2.8 (1.2-6.5)	142	3.6 (3.0-4.4)
COPD (Yes or No)	0	0.0 (0.0-0.0)	7	7.4 (3.3-16.0)	5	1.6 (0.5-4.5)	182	4.1 (3.5-4.7)
Allergy (Yes or No)	5	8.3 (3.2-19.8)	14	19.7 (11.4-31.9)	75	22.6 (18.0-27.9)	708	19.0 (17.6-20.6)
Asthma	2	1.6 (0.4-5.9)	0	0.0 (0.0-0.0)	10	3.4 (1.7-6.5)	100	2.5 (2.0-3.2)
Atopic dermatitis	1	2.1 (0.3-13.6)	1	1.1 (0.1-7.3)	18	5.1 (3.1-8.3)	112	3.3 (2.7-4.0)
Rhinitis	3	5.3 (1.5-16.9)	14	19.7 (11.4-31.9)	56	16.8 (12.8-21.8)	577	15.4 (14.1-16.8)
Thyroid disease (Yes or No)	3	3.4 (0.9-11.2)	0	0.0 (0.0-0.0)	20	3.8 (2.3-6.1)	168	3.5 (2.9-4.2)

**Variables**	**Lunch frequency (n=4,370, weighted n=19,714,846)**							
	**seldom**		**1~2times/week**		**3~4times/week**		**5~7times/week**	
	n†	Mean or % (95% CI) ‡	n†	Mean or % (95% CI)‡	n†	Mean or % (95% CI) ‡	n†	Mean or % (95% CI)‡
Viral liver disease (Yes or No)	2	3.5 (0.9-12.7)	1	0.2 (0.0-1.5)	15	5.5 (3.2-9.3)	188	4.7 (3.9-5.6)
Hepatitis B	2	3.5 (0.9-12.7)	1	0.2 (0.0-1.5)	14	5.0 (2.8-8.7)	170	4.3 (3.6-5.1)
Hepatitis C	0	0.0 (0.0-0.0)	0	0.0 (0.0-0.0)	1	0.5 (0.1-3.5)	11	0.3 (0.1-0.5)
Liver cirrhosis	0	0.0 (0.0-0.0)	0	0.0 (0.0-0.0)	0	0.0 (0.0-0.0)	12	0.3 (0.2-0.5)
2013 (enrolled year)	27	46.3 (32.0-61.3)	43	43.1 (31.2-55.8)	156	44.4 (36.2-53.0)	2,080	51.9 (46.1-57.7)
2014	37	53.7 (38.7-68.0)	42	56.9 (44.2-66.8)	167	55.6 (47.0-63.8)	1,818	48.1 (42.3-53.9)

Seldom : n = 64, weighted n = 280,106.5.
1~2times/week : n = 85, weighted n = 387,689.5.
3~4times/week : n = 323, weighted n = 1,565,213.
5~7times/week : n = 3,898, weighted n = 17,481,847.

Supplementary Table S4. Baseline characteristics of the study population by dinner frequency.

Variables	Dinner frequency (n=4,370, weighted n=19,714,846)							
	seldom		1~2times/week		3~4times/week		5~7times/week	
	n†	Mean or % (95% CI) ‡	n†	Mean or % (95% CI)‡	n†	Mean or % (95% CI) ‡	n†	Mean or % (95% CI)‡
Male sex (Yes or No)	4	25.3 (9.0-53.5)	16	26.3 (16.4-39.5)	114	38.3 (32.6-44.4)	1,670	54.6 (53.1-56.2)
Age (years)	23	40.7 (35.1-46.4)	79	36.2 (33.4-39.1)	405	36.9 (35.6-38.2)	3,863	41.8 (41.3-42.4)
Annual family income								
High	5	18.8 (6.3-44.4)	18	23.6 (14.4-36.1)	150	36.7 (31.7-42.1)	1,297	34.2 (31.5-37.1)
Medium high	6	26.2 (8.3-58.3)	30	38.1 (26.2-51.6)	112	26.1 (21.3-31.4)	1,223	32.2 (29.9-34.5)
Medium low	9	44.2 (22.0-69.0)	23	28.7 (18.5-41.6)	117	30.0 (25.0-35.5)	974	25.1 (22.9-27.5)
Low	3	10.8 (3.1-31.4)	8	9.7 (4.4-20.1)	24	6.7 (4.1-10.9)	361	8.3 (7.2-9.6)
Education								
More than college	3	12.3 (3.7-33.6)	42	55.0 (42.5-67.0)	163	41.9 (36.5-47.5)	1,529	41.8 (39.6-44.0)
High school	13	58.6 (33.6-79.8)	25	32.9 (22.0-46.0)	186	46.8 (41.2-52.4)	1,500	41.2 (39.3-43.2)
Middle school	2	5.9 (1.4-21.9)	6	6.5 (2.3-17.3)	31	6.4 (4.4-9.3)	382	8.7 (7.6-10.0)
Less than elementary school	5	23.2 (8.1-50.8)	6	5.6 (2.3-13.0)	25	4.9 (3.0-8.0)	448	8.1 (7.1-9.2)
Job status (Yes or No)	14	70.7 (51.2-84.7)	50	61.3 (48.5-72.6)	268	68.9 (63.5-73.7)	2,552	68.5 (66.8-70.2)
Marital status								
married	16	78.6 (56.9-91.0)	48	51.8 (39.4-63.9)	255	56.2 (50.6-61.6)	2,947	70.6 (68.5-72.7)
divorce or widowed	4	11.2 (3.4-30.9)	7	7.6 (3.1-17.7)	25	5.3 (3.2-8.6)	263	5.3 (4.5-6.3)
not married	3	10.3 (3.1-28.8)	24	40.6 (28.7-53.8)	124	38.3 (33.0-44.0)	648	23.9 (21.9-26.0)

**Variables**	**Dinner frequency (n=4,370, weighted n=19,714,846)**							
	**seldom**		**1~2times/week**		**3~4times/week**		**5~7times/week**	
	n†	Mean or % (95% CI) ‡	n†	Mean or % (95% CI)‡	n†	Mean or % (95% CI) ‡	n†	Mean or % (95% CI)‡
Insurance								
NHIS	20	88.1 (66.5-96.5)	75	94.0 (88.2-98.2)	393	96.8 (93.2-98.6)	3,748	97.0 (96.1-97.7)
medical aid	2	9.4 (2.2-32.0)	3	5.4 (1.5-17.8)	12	3.2 (1.4-6.8)	87	2.1 (1.5-2.8)
Personal insurance (Yes or No)	18	71.5 (47.4-87.5)	68	85.9 (73.9-92.9)	355	87.2 (83.2-90.3)	3,338	86.4 (84.8-87.9)
Smoking								
never	18	70.1 (43.0-88.0)	49	59.9 (46.7-71.8)	266	60.9 (55.1-66.5)	2,391	55.2 (53.4-56.9)
exsmoker	1	4.8 (0.7-27.9)	11	11.0 (5.8-20.0)	48	13.5 (10.0-18.0)	656	18.6 (17.2-20.1)
current	4	25.1 (9.0-53.1)	19	29.0 (18.4-42.7)	91	25.6 (20.6-31.4)	816	26.2 (24.5-28.0)
Alcohol consumption, ever	21	78.9 (48.5-93.7)	74	93.7 (84.6-97.5)	382	94.4 (91.0-96.6)	3,540	93.5 (92.6-94.3)
Alcohol consumption in last 1 year	14	58.7 (36.2-78.0)	50	71.3 (59.6-80.7)	260	68.4 (62.7-73.7)	2,168	60.7 (58.8-62.6)
EuroQol-5D (n)	23	0.952 (0.920-0.983)	79	0.976 (0.958-0.993)	405	0.967 (0.959-0.975)	3,863	0.965 (0.961-0.968)
Systolic BP (mmHg)	23	119.2 (113.4-124.9)	79	112.3 (109.1-115.5)	405	112.4 (110.8-114.1)	3,861	114.9 (114.3-115.5)
Diastolic BP (mmHg)	23	77.2 (72.6-81.7)	79	73.6 (71.3-76.0)	405	74.3 (73.0-75.6)	3,861	75.9 (75.5-76.4)
Waist circumference (cm)	23	82.0 (76.6-87.4)	79	78.4 (76.0-80.8)	405	79.5 (78.3-80.6)	3,863	80.5 (80.1-80.9)
Abdominal obesity (Yes or No)	2	17.5 (4.5-48.9)	5	5.2 (2.1-12.3)	31	9.6 (6.6-13.6)	450	14.4 (13.1-15.8)
BMI (kg/㎡)	23	24.8 (22.7-27.0)	79	23.8 (23.1-24.6)	405	23.9 (23.5-24.3)	3,863	23.7 (23.6-23.8)

**Variables**	**Dinner frequency (n=4,370, weighted n=19,714,846)**							
	**seldom**		**1~2times/week**		**3~4times/week**		**5~7times/week**	
	n†	Mean or % (95% CI) ‡	n†	Mean or % (95% CI)‡	n†	Mean or % (95% CI) ‡	n†	Mean or % (95% CI)‡
Obesity in BMI								
Normal to pre-obesity (18.5~25)	13	50.0 (26.0-74.1)	49	67.7 (55.9-77.7)	255	62.6 (56.9-68.0)	2,486	63.6 (61.8-65.4)
Normal (18.5~23)	10	40.4 (18.5-66.9)	29	37.6 (26.0-50.7)	154	37.5 (32.5-42.7)	1,580	40.3 (38.4-42.1)
Pre-obesity (23~25)	3	9.7 (2.7-29.0)	20	30.2 (19.5-43.5)	101	25.2 (20.7-30.2)	906	23.4 (21.8-25.0)
Obesity (≥25)	10	50.0 (25.9-74.0)	29	31.6 (21.8-43.4)	137	34.0 (28.8-39.5)	1,208	32.0 (30.2-33.8)
1st obesity (25~30)	9	40.6 (19.3-66.0)	24	26.2 (17.3-37.5)	116	28.9 (24.0-34.3)	1,048	27.3 (25.7-29.0)
2nd obesity (30~35)	1	9.4 (1.3-44.4)	5	5.4 (1.9-14.7)	19	4.8 (2.8-8.0)	143	4.2 (3.5-5.1)
3rd obesity (≥35)	0	0.0 (0.0-0.0)	0	0.0 (0.0-0.0)	2	0.3 (0.1-1.2)	17	0.4 (0.2-0.7)
Under weighted (<18.5)	0	0.0 (0.0-0.0)	1	0.7 (0.1-4.6)	13	3.4 (1.9-6.1)	169	4.4 (3.7-5.2)
Exercise (Yes or No)	9	48.0 (24.1-72.9)	43	55.9 (43.1-67.9)	193	47.6 (42.2-53.1)	1,757	48.5 (46.4-50.6)
More than 150 mins/week	8	39.9 (17.5-67.5)	40	50.8 (38.1-63.3)	144	36.3 (31.4-41.5)	1,281	35.8 (33.9-37.9)
Physical activity (MET-minutes/week)	23	3,066 (28-6,105)	79	2,417 (1,571-3,263)	405	1,808 (1,563-2,053)	3,863	1,851 (1,740-1,962)
High (≥3000)	5	30.7 (13.6-55.6)	16	22.1 (12.9-35.2)	68	19.4 (15.4-24.2)	656	18.4 (16.9-20.0)
Moderate (600-3000)	7	25.1 (11.8-45.8)	31	39.4 (27.7-52.5)	195	45.2 (39.4-51.2)	1,642	43.7 (41.8-45.6)
Low (<600)	11	44.2 (22.2-68.8)	32	38.5 (27.0-51.3)	142	35.3 (30.4-40.6)	1,565	37.9 (35.9-39.9)
Serum creatinine (mg/dL)	23	0.800 (0.693-0.907)	79	0.769 (0.738-0.799)	405	0.813 (0.792-0.833)	3,863	0.855 (0.846-0.864)
MDRD eGFR (mL/min/1.73㎡)	23	92.2 (82.6-101.9)	79	95.7 (92.5-98.9)	405	93.5 (91.8-95.1)	3,863	90.5 (89.9-91.1)
CKD-EPI eGFR (mL/min/1.73㎡)	23	100.7 (91.8-109.6)	79	105.8 (102.7-108.8)	405	103.3 (101.8-104.8)	3,863	99.3 (98.7-100.0)

Variables	Dinner frequency (n=4,370, weighted n=19,714,846)							
	seldom		1~2times/week		3~4times/week		5~7times/week	
	n†	Mean or % (95% CI) ‡	n†	Mean or % (95% CI)‡	n†	Mean or % (95% CI) ‡	n†	Mean or % (95% CI)‡
Random urine albumin (ug/mL)	23	27.7 (-0.4-55.8)	79	11.3 (5.5-17.0)	405	21.9 (10.9-33.0)	3,863	21.8 (17.2-26.4)
Random urine creatinine (mg/dL)	23	137.9 (114.3-161.6)	79	145.8 (127.8-163.8)	405	146.2 (146.5-155.9)	3,863	156.8 (153.2-160.4)
Random urine ACR (mg/gCr)	23	19.7 (1.2-38.3)	79	10.5 (4.1-16.8)	405	22.1 (14.0-30.2)	3,863	25.5 (14.9-36.1)
CKD (Yes or No)	3	16.3 (4.2-46.3)	5	8.2 (3.1-20.0)	43	12.5 (9.0-17.2)	361	9.3 (8.2-10.4)
Fasting glucose (mg/dL)	23	96.3 (91.0-101.5)	79	92.2 (88.8-95.6)	405	95.1 (92.7-97.6)	3,863	97.1 (96.4-97.8)
HbA1c (%)	23	5.568 (5.333-5.802)	79	5.533 (5.395-5.671)	405	5.610 (5.531-5.689)	3,863	5.701 (5.674-5.728)
Total cholesterol (mg/dL)	23	189.2 (171.1-207.4)	79	183.0 (175.4-190.6)	405	186.2 (182.3-190.1)	3,863	187.9 (186.5-189.2)
HDL (mg/dL)	23	49.0 (43.8-54.2)	79	56.1 (53.0-59.3)	405	53.1 (51.9-54.3)	3,863	51.1 (50.7-51.5)
Triglyceride (mg/dL)	23	133.3 (94.0-172.7)	79	113.1 (92.7-133.5)	405	126.9 (115.5-138.3)	3,863	137.9 (133.3-142.5)
LDL (mg/dL)	23	117.4 (102.6-132.1)	79	105.9 (99.8-112.0)	405	110.9 (107.4-114.3)	3,863	112.1 (111.0-113.3)
Hemoglobin (g/dL)	23	13.18 (11.78-14.58)	79	13.70 (13.33-14.07)	405	14.07 (13.88-14.25)	3,863	14.41 (14.35-14.47)
Random urine sodium (mmol/L)	12	91.2 (47.5-134.8)	42	113.0 (88.5-137.5)	202	107.0 (100.1-113.8)	1,808	114.9 (112.2-117.7)
Vitamin D (ng/mL)	8	19.4 (13.5-25.4)	42	14.0 (12.4-15.7)	201	15.2 (14.3-16.0)	1,705	16.4 (16.0-16.9)
Daily Calorie intake (kcal/day)	23	1,702 (1,275-2,128)	79	1,799 (1,596-2,002)	405	2,024 (1,909-2,139)	3,863	2,226 (2,193-2,258)
Daily protein intake (g/day)	23	56.1 (43.8-68.5)	79	60.2 (52.2-68.2)	405	69.1 (64.0-74.1)	3,863	72.6 (71.3-74.0)
Daily fat intake (g/day)	23	36.6 (27.8-45.3)	79	40.5 (33.9-47.1)	405	46.4 (42.7-50.0)	3,863	44.8 (43.8-45.9)
Daily cholesterol intake (g/day)	23	200.2 (153.4-247.0)	79	253.3 (204.5-302.2)	405	274.7 (253.0-296.3)	3,863	271.4(264.3-278.4)
Daily carbohydrate intake (g/day)	23	242.2 (203.7-280.7)	79	267.6 (236.8-298.3)	405	300.0 (285.7-314.3)	3,863	347.9 (343.4-352.5)

**Variables**	**Dinner frequency (n=4,370, weighted n=19,714,846)**							
	**seldom**		**1~2times/week**		**3~4times/week**		**5~7times/week**	
	n†	Mean or % (95% CI) ‡	n†	Mean or % (95% CI)‡	n†	Mean or % (95% CI) ‡	n†	Mean or % (95% CI)‡
Daily calcium intake (mg/day)	23	450.1 (301.1-599.1)	79	496.2 (422.8-569.6)	405	499.4 (468.0-530.7)	3,863	519.5 (510.2-528.8)
Daily phosphorus intake (mg/day)	23	916 (655-1,178)	79	954 (827-1,082)	405	1,029 (967-1,091)	3,863	1,099 (1,082-1,117)
Daily sodium intake (mg/day)	23	3,008 (2,063-3,952)	79	3,042 (2,581-3,502)	405	3,434 (3,193-3,676)	3,863	3,606 (3,533-3,680)
Daily potassium intake (mg/day)	23	2,937 (2,015-3,859)	79	2,714 (2,242-3,186)	405	2,817 (2,650-2,984)	3,863	3,025 (2,974-3,077)
Daily sodium potassium intake ratio (n)	23	1.034 (0.902-1.167)	79	1.163 (1.084-1.243)	405	1.234 (1.199-1.270)	3,863	1.192 (1.180-1.205)
Body weight change								
no change	13	66.1 (42.7-83.6)	31	41.5 (29.3-54.9)	196	47.7 (42.2-53.3)	2,463	62.8 (60.9-64.7)
increase	2	4.5 (1.0-17.1)	34	39.3 (27.7-52.3)	144	35.3 (30.2-40.8)	906	23.7 (22.1-25.4)
decrease	8	29.5 (13.4-53.0)	14	19.1 (10.9-31.4)	64	16.9 (12.9-21.8)	494	13.5 (12.1-15.0)
Body weight control								
Maintain or not try	10	42.4 (23.8-63.4)	20	21.8 (13.7-32.7)	131	32.4 (27.3-37.9)	1,947	50.5 (48.7-52.2)
increase	0	0.0 (0.0-0.0)	0	0.0 (0.0-0.0)	13	4.3 (2.1-8.4)	232	6.8 (5.9-7.8)
decrease	13	57.6 (36.6-76.2)	59	78.2 (67.3-86.3)	261	63.3 (57.8-68.6)	1,684	42.8 (41.1-44.5)
Diet (Yes or No)	15	64.8 (39.4-83.9)	59	78.2 (67.3-86.3)	282	67.9 (62.3-73.0)	1,809	46.2 (44.4-47.9)
Diet by fasting	11	33.5 (18.7-52.5)	56	73.7 (61.7-82.9)	257	61.2 (56.0-66.1)	1,560	39.5 (37.8-41.3)
Diet for underlying disease	1	2.9 (0.4-18.9)	3	5.6 (1.5-18.9)	10	2.6 (1.3-5.3)	185	4.1 (3.5-4.9)
Hypertension (Yes or No)	9	31.4 (13.9-56.4)	13	13.6 (7.3-24.1)	63	15.5 (11.8-20.2)	820	19.4 (17.9-21.0)
Diabetes (Yes or No)	1	2.6 (0.4-17.3)	4	3.8 (1.1-12.1)	26	4.9 (3.2-7.5)	372	8.4 (7.4-9.4)

Variables	Dinner frequency (n=4,370, weighted n=19,714,846)							
	seldom		1~2times/week		3~4times/week		5~7times/week	
	n†	Mean or % (95% CI) ‡	n†	Mean or % (95% CI)‡	n†	Mean or % (95% CI) ‡	n†	Mean or % (95% CI)‡
Old Coronary arterial disease (Yes or No)	0	0.0 (0.0-0.0)	1	0.4 (0.1-3.1)	6	1.0 (0.4-2.5)	43	0.8 (0.6-1.2)
Myocardial infarction	0	0.0 (0.0-0.0)	0	0.0 (0.0-0.0)	2	0.2 (0.0-0.7)	12	0.2 (0.1-0.4)
Angina	0	0.0 (0.0-0.0)	1	0.4 (0.1-3.1)	5	0.9 (0.3-2.3)	34	0.7 (0.5-1.0)
Old cerebrovascular accident (Yes or No)	0	0.0 (0.0-0.0)	1	0.6 (0.1-4.6)	1	0.1 (0.0-0.5)	51	1.1 (0.8-1.6)
Dyslipidemia, (Yes or No)	11	47.4 (24.0-72.0)	19	19.1 (11.6-29.7)	124	29.2 (24.4-34.4)	1,411	36.1 (34.4-37.9)
Malignancy (Yes or No)	0	0.0 (0.0-0.0)	2	1.8 (0.4-7.3)	9	2.2 (1.0-4.6)	102	2.0 (1.6-2.5)
Depression (Yes or No)	1	10.4 (1.7-44.0)	11	14.2 (7.4-25.5)	34	8.0 (5.4-11.9)	229	5.5 (4.7-6.4)
PHQ-9 score (n)	12	2.46 (0.82-4.11)	42	5.19 (3.37-7.00)	202	3.38 (2.73-4.02)	1,801	2.56 (2.37-2.75)
Arthritis (Yes or No)	2	15.7 (4.2-44.0)	6	4.4 (1.9-10.1)	28	5.3 (3.5-8.1)	305	6.1 (5.4-6.9)
Osteoarthritis	2	15.7 (4.2-44.0)	5	3.8 (1.5-9.4)	22	3.8 (2.3-6.0)	261	5.1 (4.5-5.9)
Rheumatoid arthritis	0	0.0 (0.0-0.0)	1	0.6 (0.1-4.4)	7	1.7 (0.7-4.0)	48	1.1 (0.8-1.5)
Tuberculosis (Yes or No)	0	0.0 (0.0-0.0)	1	0.9 (0.1-6.2)	6	1.9 (0.8-4.3)	150	3.9 (3.2-4.7)
COPD (Yes or No)	1	2.8 (0.4-18.1)	4	4.1 (1.6-10.4)	8	2.6 (1.3-5.2)	181	4.0 (3.4-4.7)
Allergy (Yes or No)	5	23.2 (9.2-47.4)	17	23.6 (14.1-36.9)	77	19.8 (15.9-24.4)	703	19.0 (17.5-20.5)
Asthma	0	0.0 (0.0-0.0)	3	3.2 (1.0-9.6)	8	2.5 (1.2-5.3)	101	2.5 (2.0-3.2)
Atopic dermatitis	2	8.8 (2.1-30.5)	6	10.3 (4.3-23.0)	20	4.6 (2.8-7.4)	104	3.1 (2.5-3.8)
Rhinitis	4	18.4 (6.2-43.7)	12	16.1 (8.1-29.4)	64	15.8 (12.3-20.2)	570	15.4 (14.0-16.8)
Thyroid disease (Yes or No)	2	4.5 (1.0-17.1)	6	5.0 (2.2-10.9)	15	3.0 (1.7-5.2)	168	3.5 (2.9-4.2)

Variables	Dinner frequency (n=4,370, weighted n=19,714,846)							
	seldom		1~2times/week		3~4times/week		5~7times/week	
	n†	Mean or % (95% CI) ‡	n†	Mean or % (95% CI)‡	n†	Mean or % (95% CI) ‡	n†	Mean or % (95% CI)‡
Viral liver disease (Yes or No)	3	10.7 (3.7-27.1)	1	1.1 (0.2-7.8)	12	3.1 (1.6-5.8)	190	4.9 (4.1-5.8)
Hepatitis B	2	5.4 (1.3-20.5)	1	1.1 (0.2-7.8)	12	3.1 (1.6-5.8)	172	4.4 (3.7-5.3)
Hepatitis C	1	5.3 (0.9-25.4)	0	0.0 (0.0-0.0)	0	0.0 (0.0-0.0)	11	0.3 (0.1-0.5)
Liver cirrhosis	0	0.0 (0.0-0.0)	0	0.0 (0.0-0.0)	0	0.0 (0.0-0.0)	12	0.3 (0.2-0.5)
2013 (enrolled year)	11	48.9 (25.1-73.3)	37	45.0 (32.4-58.3)	203	48.3 (40.4-56.3)	2,055	51.5 (45.7-57.3)
2014	12	51.1 (26.7-74.9)	42	55.0 (41.7-67.6)	202	51.7 (43.7-59.6)	1,808	48.5 (42.7-54.3)

Seldom : n = 23, weighted n = 100,928.
1~2times/week : n = 79, weighted n = 334,137.5.
3~4times/week : n = 405, weighted n = 1,874,468.
5~7times/week : n = 3,863, weighted n = 17,405,312.5.

Supplementary Table S5. Complex samples multivariate logistic regression for analyzing the prevalence of chronic kidney disease (CKD) by lunch frequency.

Variables	Unadjusted OR (95% CI)	Model 1 (95% CI)	Model 2 (95% CI)	Model 3 (95% CI)
Lunch (Ref.: 5~7times/week)				
3~4times/week	1.231 (0.813-1.864)	1.362 (0.902-2.057)	1.413 (0.930-2.146)	1.327 (0.868-2.027)
1~2times/week	1.158 (0.514-2.609)	1.269 (0.562-2.866)	1.497 (0.653-3.432)	1.330 (0.562-3.152)
seldom	1.540 (0.601-3.945)	1.631 (0.626-4.251)	1.652 (0.612-4.459)	1.596 (0.584-4.365)
Male gender (Yes or No)		1.409 (1.119-1.773)	1.317 (0.902-1.924)	1.834 (1.204-2.794)
Age (per year)		1.014 (1.004-1.024)	0.996 (0.985-1.007)	0.988 (0.977-1.000)
BMI (Ref.: normal, 18.5-25.0 Kg/㎡)				
Obese (≥25.0kg/㎡)			1.261 (0.976-1.630)	1.272 (0.981-1.649)
Underweight (<18.5 kg/㎡)			1.899 (1.056-3.413)	2.025 (1.113-3.684)
Physical activity (Ref.: high, ≥3000)				
Moderate (600-2999)			1.256 (0.902-1.751)	1.235 (0.885-1.723)
Low (<600)			1.062 (0.759-1.486)	1.008 (0.718-1.417)
Hypertension (Yes or No)			1.997 (1.496-2.665)	2.049 (1.535-2.734)
Diabetes (Yes or No)			3.118 (2.250-4.322)	1.856 (1.185-2.908)
Old CAD (Yes or No)			1.389(0.646-2.986)	1.665 (0.781-3.549)
Old CVA (Yes or No)			2.103 (1.172-3.773)	2.227 (1.247-3.977)
Smoking (Ref.: never)				
Exsmoker			0.925 (0.612-1.397)	0.921 (0.611-1.390)
Current smoker			0.875 (0.584-1.310)	0.897 (0.595-1.351)
Hemoglobin (g/dL)				0.908 (0.809-1.018)
Fasting glucose (mg/dL)				1.011 (1.005-1.017)
HDL (mg/dL)				1.004 (0.993-1.015)
LDL (mg/dL)				1.004 (1.000-1.007)
Daily calorie intake (g/day, log transformed)				0.751 (0.603-0.937)

Abbreviation: OR, odd ratio; Ref., reference; CAD, coronary artery disease; CVA, cerebrovascular accident.
Model 1 : gender, age.
Model 2 : Model 1 + BMI, physical activity(MET), hypertension, diabetes, old CAD, old CVA, smoking.
Model 3 : Model 2 + hemoglobin, fasting glucose, HDL, LDL, daily calorie intake.

Supplementary Table S6. Complex samples multivariate logistic regression for analyzing the prevalence of CKD by dinner frequency.

Variables	Unadjusted OR (95% CI)	Model 1 (95% CI)	Model 2 (95% CI)	Model 3 (95% CI)
Dinner (Ref.: 5~7times/week)				
3~4times/week	1.404 (0.944-2.089)	1.605 (1.074-2.398)	1.630 (1.098-2.419)	1.517 (1.006-2.286)
1~2times/week	0.875 (0.309-2.481)	1.052 (0.369-2.997)	1.106 (0.380-3.226)	1.007 (0.329-3.085)
seldom	1.912 (0.430-8.497)	2.176 (0.458-10.334)	2.236 (0.469-10.659)	1.854 (0.395-8.708)
Male gender (Yes or No)		1.438 (1.145-1.805)	1.339 (0.927-1.934)	1.852 (1.217-2.819)
Age (per year)		1.014 (1.004-1.024)	0.997 (0.986-1.008)	0.989 (0.978-1.001)
BMI (Ref.: normal, 18.5-25.0 Kg/㎡)				
Obese (≥25.0kg/㎡)			1.256 (0.969-1.629)	1.269 (0.976-1.649)
Underweight (<18.5 kg/㎡)			1.972 (1.099-3.537)	2.092 (1.151-3.801)
Physical activity (Ref.: high, ≥3000)				
Moderate (600-2999)			1.266 (0.908-1.765)	1.241 (0.890-1.734)
Low (<600)			1.082 (0.772-1.515)	1.023 (0.725-1.442)
Hypertension (Yes or No)			1.958 (1.472-2.604)	2.016 (1.516-2.682)
Diabetes (Yes or No)			3.145 (2.265-4.367)	1.897 (1.214-2.966)
Old CAD (Yes or No)			1.362 (0.642-2.892)	1.631 (0.775-3.434)
Old CVA (Yes or No)			2.167 (1.200-3.912)	2.279 (1.270-4.089)
Smoking (Ref.: never)				
Exsmoker			0.927 (0.616-1.394)	0.923 (0.614-1.387)
Current smoker			0.886 (0.592-1.326)	0.907 (0.603-1.365)
Hemoglobin (g/dL)				0.909 (0.811-1.019)
Fasting glucose (mg/dL)				1.011 (1.005-1.017)
HDL (mg/dL)				1.004 (0.993-1.015)
LDL (mg/dL)				1.004 (1.000-1.007)
Daily calorie intake (g/day, log transformed)				0.753 (0.597-0.948)

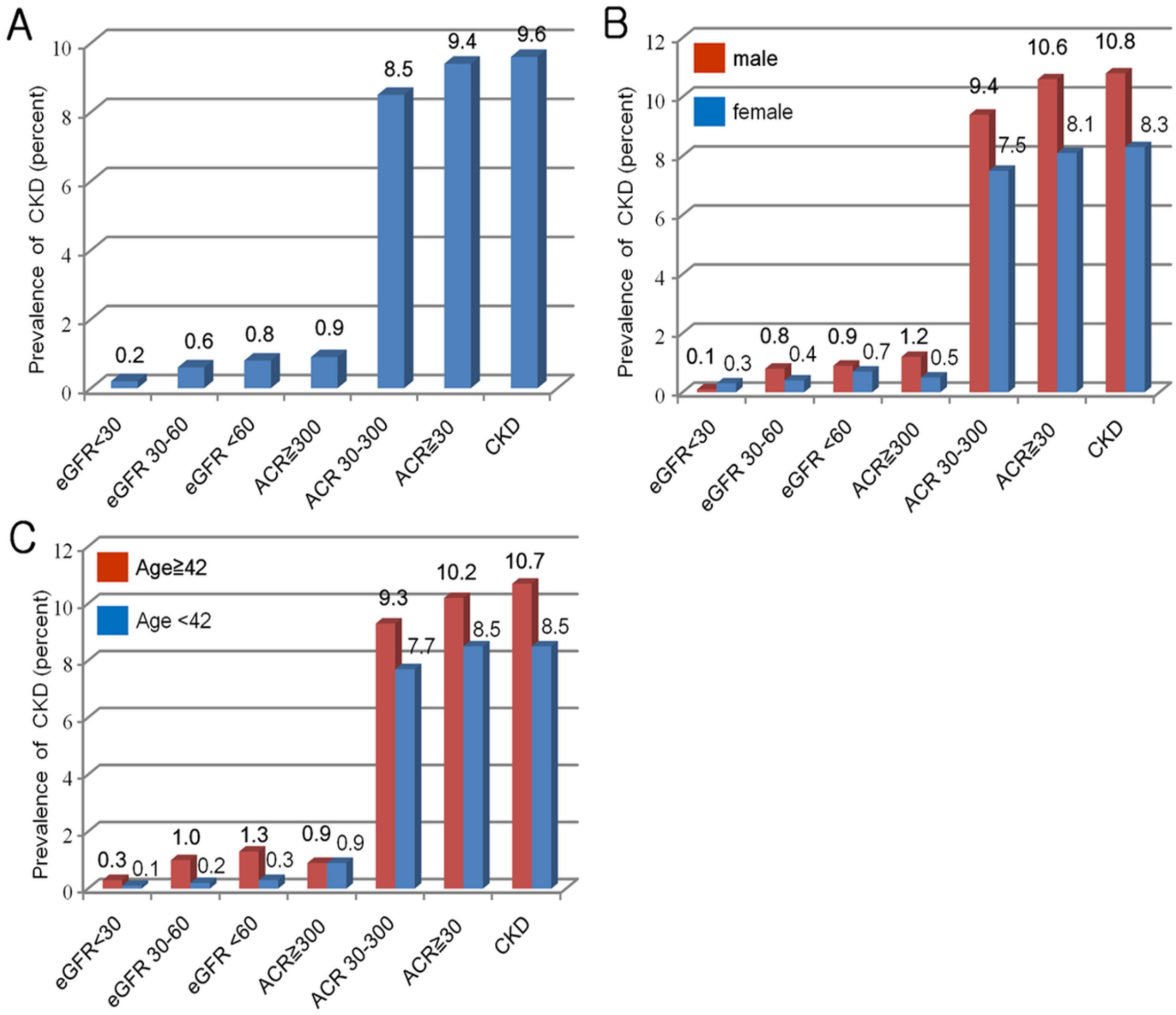

Supplementary Figure S1. (**A**) Prevalence of chronic kidney disease (CKD) in the study population; (**B**) Prevalence of CKD by gender; and (**C**) Prevalence of CKD by the median age of 42 years. Abbreviation: eGFR, estimated glomerular filtration rate; ACR, albumin creatinine ratio.

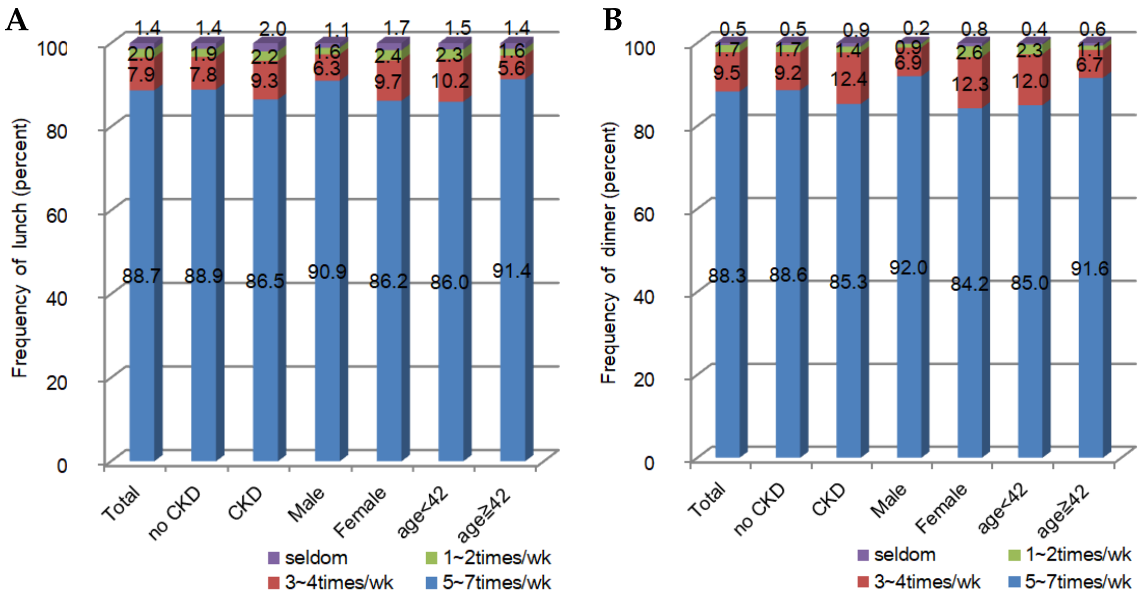

Supplementary Figure 2. (**A**) Frequency of lunch intake by CKD, gender and age, (**B**) Frequency of dinner intake by CKD, gender, and age.

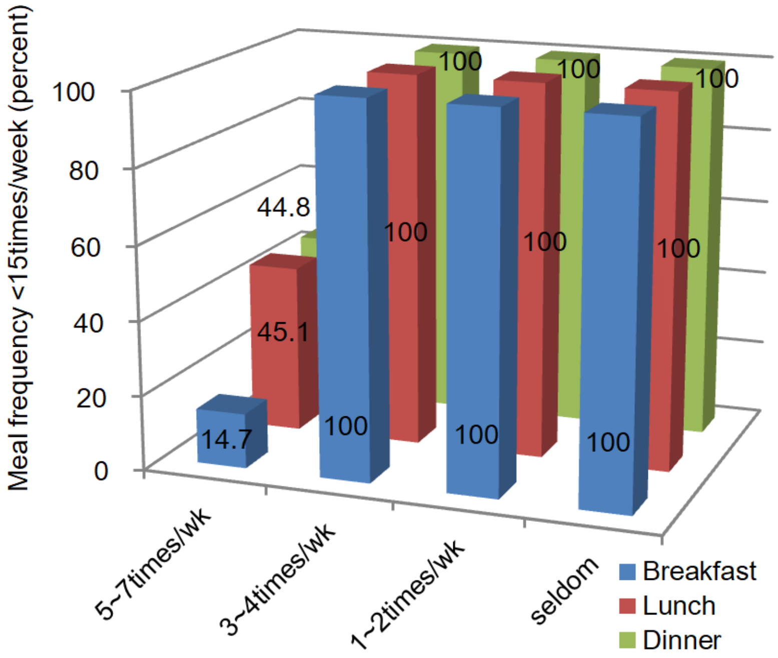

Supplementary Figure 3. Association of meal frequency with the frequency of breakfast, lunch, and dinner.
